# Continuity of Channel Parameters and Operations under Various DMC Topologies †

**DOI:** 10.3390/e20050330

**Published:** 2018-04-30

**Authors:** Rajai Nasser

**Affiliations:** École Polytechnique Fédérale de Lausanne, Route Cantonale, 1015 Lausanne, Switzerland; rajai.nasser@epfl.ch; Tel.: +41-76-730-8363

**Keywords:** continuity, channel capacity, topology

## Abstract

We study the continuity of many channel parameters and operations under various topologies on the space of equivalent discrete memoryless channels (DMC). We show that mutual information, channel capacity, Bhattacharyya parameter, probability of error of a fixed code and optimal probability of error for a given code rate and block length are continuous under various DMC topologies. We also show that channel operations such as sums, products, interpolations and Arıkan-style transformations are continuous.

## 1. Introduction

This paper is an extended version of our paper that is published in the International Symposium on Information Theory 2017 (ISIT 2017) [1].

Let X and Y be two finite sets, and let *W* be a fixed channel with input alphabet X and output alphabet Y. It is well known that the input-output mutual information is continuous on the simplex of input probability distributions. Many other parameters that depend on the input probability distribution were shown to be continuous on the simplex in [2].

Polyanskiy studied in [3] the continuity of the Neyman–Pearson function for a binary hypothesis test that arises in the analysis of channel codes. He showed that for arbitrary input and output alphabets, this function is continuous in the input distribution in the total variation topology. He also showed that under some regularity assumptions, this function is continuous in the weak-∗ topology.

If X and Y are finite sets, the space of channels with input alphabet X and output alphabet Y can naturally be endowed with the topology of the Euclidean metric, or any other equivalent metric. It is well known that the channel capacity is continuous in this topology. If X and Y are arbitrary, one can construct a topology on the space of channels using the weak-∗ topology on the output alphabet. It was shown in [4] that the capacity is lower semi-continuous in this topology.

The continuity results that are mentioned in the previous paragraph do not take into account “equivalence” between channels. Two channels are said to be equivalent if they are degraded from each other. This means that each channel can be simulated from the other by local operations at the receiver. Two channels that are degraded from each other are completely equivalent from an operational point of view: both channels have exactly the same probability of error under optimal decoding for any fixed code. Moreover, any sub-optimal decoder for one channel can be transformed to a sub-optimal decoder for the other channel with the same probability of error and essentially the same computational complexity. This is why it makes sense, from an information-theoretic point of view, to identify equivalent channels and consider them as one point in the space of “equivalent channels”.

In [5], equivalent binary-input channels were identified with their *L*-density (i.e., the density of log-likelihood ratios). The space of equivalent binary-input channels was endowed with the topology of convergence in distribution of *L*-densities. Since the symmetric capacity (the symmetric capacity is the input-output mutual information with uniformly-distributed input) and the Bhattacharyya parameter can be written as an integral of a continuous function with respect to the *L*-density [5], it immediately follows that these parameters are continuous in the *L*-density topology.

In [6], many topologies were constructed for the space of equivalent channels sharing a fixed input alphabet. In this paper, we study the continuity of many channel parameters and operations under these topologies. The continuity of channel parameters and operations might be helpful in the following two problems:

- If a parameter (such as the optimal probability of error of a given code) is difficult to compute for a channel *W*, one can approximate it by computing the same parameter for a sequence of channels ${\left(W_{n}\right)}_{n\geq 0}$ that converges to *W* in some topology where the parameter is continuous.
- The study of the robustness of a communication system against the imperfect specification of the channel.

In Section 2, we introduce the preliminaries for this paper. In Section 3, we recall the main results of [6] that we need here. In Section 4, we introduce the channel parameters and operations that we investigate in this paper. In Section 5, we study the continuity of these parameters and operations in the quotient topology of the space of equivalent channels with fixed input and output alphabets. The continuity in the strong topology of the space of equivalent channels sharing the same input alphabet is studied in Section 6. Finally, the continuity in the noisiness/weak-∗ and the total variation topologies is studied in Section 7.

## 2. Preliminaries

We assume that the reader is familiar with the basic concepts of General Topology. The main concepts and theorems that we need can be found in the Preliminaries Section of [6].

### 2.1. Set-Theoretic Notations

For every integer n≥1, we denote the set {1,…,n} as [n].

The set of mappings from a set *A* to a set *B* is denoted as $B^{A}$.

Let *A* be a subset of *B*. The indicator mapping ${𝟙}_{A,B}:B\to \left\{0,1\right\}$ of *A* in *B* is defined as:

$${𝟙}_{A,B}\left(x\right)={𝟙}_{x\in A}=\left\{\begin{array}{cc} 1\; & \mathrm{if}\;x\in A,\\ 0\; & \mathrm{otherwise}. \end{array}\right.$$

If the superset *B* is clear from the context, we simply write ${𝟙}_{A}$ to denote the indicator mapping of *A* in *B*.

The power set of *B* is the set of subsets of *B*. Since every subset of *B* can be identified with its indicator mapping, we denote the power set of *B* as ${\left\{0,1\right\}}^{B}=2^{B}$.

Let ${\left(A_{i}\right)}_{i\in I}$ be a collection of arbitrary sets indexed by *I*. The disjoint union of ${\left(A_{i}\right)}_{i\in I}$ is defined as $\underset{i\in I}{∐}A_{i}=\underset{i\in I}{⋃}\left(A_{i}\times \left\{i\right\}\right)$. For every i∈I, the *i*-th-canonical injection is the mapping $\phi_{i}:A_{i}\to \underset{j\in I}{∐}A_{j}$ defined as $\phi_{i}\left(x_{i}\right)=\left(x_{i},i\right)$. If no confusions can arise, we can identify $A_{i}$ with $A_{i}\times \left\{i\right\}$ through the canonical injection. Therefore, we can see $A_{i}$ as a subset of $\underset{j\in I}{∐}A_{j}$ for every i∈I.

Let *R* be an equivalence relation on a set *T*. For every x∈T, the set $\hat{x}=\left\{y\in T:\;xRy\right\}$ is the *R*-equivalence class of *x*. The collection of *R*-equivalence classes, which we denote as T/R, forms a partition of *T*, and it is called the quotient space of *T* by *R*. The mapping ${\mathrm{Proj}}_{R}:T\to T/R$ defined as ${\mathrm{Proj}}_{R}\left(x\right)=\hat{x}$ for every x∈T is the projection mapping onto T/R.

### 2.2. Topological Notations

A topological space (T,U) is said to be contractible to $x_{0}\in T$ if there exists a continuous mapping H:T×[0,1]→T such that H(x,0)=x and $H\left(x,1\right)=x_{0}$ for every x∈T, where [0,1] is endowed with the Euclidean topology. (T,U) is strongly contractible to $x_{0}\in T$ if we also have $H\left(x_{0},t\right)=x_{0}$ for every t∈[0,1].

Intuitively, *T* is contractible if it can be “continuously shrinked” to a single point $x_{0}$. If this “continuous shrinking” can be done without moving $x_{0}$, *T* is strongly contractible.

Note that contractibility is a very strong notion of connectedness: every contractible space is path-connected and simply connected. Moreover, all its homotopy, homology and cohomology groups of order ≥1 are zero.

Let ${\left\{\left(T_{i},U_{i}\right)\right\}}_{i\in I}$ be a collection of topological spaces indexed by *I*. The product topology on $\prod_{i\in I}T_{i}$ is denoted by $\underset{i\in I}{⨂}U_{i}$. The disjoint union topology on $\underset{i\in I}{∐}T_{i}$ is denoted by $\underset{i\in I}{⨁}U_{i}$.

The following lemma is useful to show the continuity of many functions.

**Lemma** **1.** *Let (S,V) and (T,U) be two compact topological spaces, and let f:S×T→R be a continuous function on S×T. For every s∈S and every ϵ>0, there exists a neighborhood $V_{s}$ of s such that for every $s^{\prime }\in V_{s}$, we have:*

$$\underset{t\in T}{\sup}\left|f\left(s^{\prime },t\right)-f\left(s,t\right)\right|\leq \epsilon .$$

**Proof.**  See Appendix A. ☐

### 2.3. Quotient Topology

Let (T,U) be a topological space, and let *R* be an equivalence relation on *T*. The quotient topology on T/R is the finest topology that makes the projection mapping ${\mathrm{Proj}}_{R}$ continuous. It is given by:

$$U/R=\left\{\hat{U}\subset T/R:\;{\mathrm{Proj}}_{R}^{-1}\left(\hat{U}\right)\in U\right\}.$$

**Lemma** **2.** *Let f:T→S be a continuous mapping from (T,U) to (S,V). If $f\left(x\right)=f\left(x^{\prime }\right)$ for every $x,x^{\prime }\in T$ satisfying $xRx^{\prime }$, then we can define a transcendent mapping f:T/R→S such that $f\left(\hat{x}\right)=f\left(x^{\prime }\right)$ for any $x^{\prime }\in \hat{x}$. f is well defined on T/R. Moreover, f is a continuous mapping from (T/R,U/R) to (S,V).*

Let (T,U) and (S,V) be two topological spaces, and let *R* be an equivalence relation on *T*. Consider the equivalence relation $R^{\prime }$ on T×S defined as $\left(x_{1},y_{1}\right)R^{\prime }\left(x_{2},y_{2}\right)$ if and only if $x_{1}Rx_{2}$ and $y_{1}=y_{2}$. A natural question to ask is whether the canonical bijection between $\left((T/R)\times S,(U/R)⊗V\right)$ and $\left(\left(T\times S\right)/R^{\prime },\left(U⊗V\right)/R^{\prime }\right)$ is a homeomorphism. It turns out that this is not the case in general. The following theorem, which is widely used in Algebraic Topology, provides a sufficient condition:

**Theorem** **1.** *[7] If (S,V) is locally compact and Hausdorff, then the canonical bijection between ((T/R)×S, (U/R)⊗V) and $\left(\left(T\times S\right)/R^{\prime },\left(U⊗V\right)/R^{\prime }\right)$ is a homeomorphism.*

**Corollary** **1.** *Let (T,U) and (S,V) be two topological spaces, and let $R_{T}$ and $R_{S}$ be two equivalence relations on T and S, respectively. Define the equivalence relation R on T×S as $\left(x_{1},y_{1}\right)R\left(x_{2},y_{2}\right)$ if and only if $x_{1}R_{T}x_{2}$ and $y_{1}R_{S}y_{2}$. If (S,V) and $\left(T/R_{T},U/R_{T}\right)$ are locally compact and Hausdorff, then the canonical bijection between $\left(\left(T/R_{T}\right)\times \left(S/R_{S}\right),\left(U/R_{T}\right)⊗\left(V/R_{S}\right)\right)$ and $\left((T\times S)/R,(U⊗V)/R\right)$ is a homeomorphism.*

**Proof.**  We just need to apply Theorem 1 twice. Define the equivalence relation $R_{T}^{\prime }$ on T×S as follows: $\left(x_{1},y_{1}\right)R_{T}^{\prime }\left(x_{2},y_{2}\right)$ if and only if $x_{1}R_{T}x_{2}$ and $y_{1}=y_{2}$. Since (S,V) is locally compact and Hausdorff, Theorem 1 implies that the canonical bijection from $\left(\left(T/R_{T}\right)\times S,\left(U/R_{T}\right)⊗V\right)$ to $\left(\left(T\times S\right)/R_{T}^{\prime },\left(U⊗V\right)/R_{T}^{\prime }\right)$ is a homeomorphism. Let us identify these two spaces through the canonical bijection.

Now, define the equivalence relation $R_{S}^{\prime }$ on $(T/R_{T})\times S$ as follows: $\left({\hat{x}}_{1},y_{1}\right)R_{S}^{\prime }\left({\hat{x}}_{2},y_{2}\right)$ if and only if ${\hat{x}}_{1}={\hat{x}}_{2}$ and $y_{1}R_{S}y_{2}$. Since $\left(T/R_{T},U/R_{T}\right)$ is locally compact and Hausdorff, Theorem 1 implies that the canonical bijection from $\left(\left(T/R_{T}\right)\times \left(S/R_{S}\right),\left(U/R_{T}\right)⊗\left(V/R_{S}\right)\right)$ to $\left(\left(\left(T/R_{T}\right)\times S\right)/R_{S}^{\prime },\left(\left(U/R_{T}\right)⊗V\right)/R_{S}^{\prime }\right)$ is a homeomorphism.

Since we identified $\left(\left(T/R_{T}\right)\times S,\left(U/R_{T}\right)⊗V\right)$ and $\left(\left(T\times S\right)/R_{T}^{\prime },\left(U⊗V\right)/R_{T}^{\prime }\right)$ through the canonical bijection (which is a homeomorphism), $R_{S}^{\prime }$ can be seen as an equivalence relation on $\left(\left(T\times S\right)/R_{T}^{\prime },\left(U⊗V\right)/R_{T}^{\prime }\right)$. It is easy to see that the canonical bijection from $\left(\left(\left(T\times S\right)/R_{T}^{\prime }\right)/R_{S}^{\prime },\left(\left(U⊗V\right)/R_{T}^{\prime }\right)/R_{S}^{\prime }\right)$ to $\left((T\times S)/R,(U⊗V)/R\right)$ is a homeomorphism. We conclude that the canonical bijection from $\left(\left(T/R_{T}\right)\times \left(S/R_{S}\right),\left(U/R_{T}\right)⊗\left(V/R_{S}\right)\right)$ to $\left((T\times S)/R,(U⊗V)/R\right)$ is a homeomorphism. ☐

### 2.4. Measure-Theoretic Notations

If (M,Σ) is a measurable space, we denote the set of probability measures on (M,Σ) as P(M,Σ). If the σ-algebra Σ is known from the context, we simply write P(M) to denote the set of probability measures.

If P∈P(M,Σ) and {x} is a measurable singleton, we simply write P(x) to denote P({x}).

For every $P_{1},P_{2}\in P\left(M,\Sigma \right)$, the total variation distance between $P_{1}$ and $P_{2}$ is defined as:

$$∥P_{1}-P_{2}{∥}_{TV}=\underset{A\in \Sigma }{\sup}\left|P_{1}\left(A\right)-P_{2}\left(A\right)\right|.$$

- The push-forward probability measure:
  Let *P* be a probability measure on (M,Σ), and let $f:M\to M^{\prime }$ be a measurable mapping from (M,Σ) to another measurable space $\left(M^{\prime },\Sigma^{\prime }\right)$. The push-forward probability measure of *P* by *f* is the probability measure $f_{\#}P$ on $\left(M^{\prime },\Sigma^{\prime }\right)$ defined as $\left(f_{\#}P\right)\left(A^{\prime }\right)=P\left(f^{-1}\left(A^{\prime }\right)\right)$ for every $A^{\prime }\in \Sigma^{\prime }$.
  A measurable mapping $g:M^{\prime }\to R$ is integrable with respect to $f_{\#}P$ if and only if g∘f is integrable with respect to *P*. Moreover,
  $$\int_{M^{\prime }}g\cdot d\left(f_{\#}P\right)=\int_{M}\left(g\circ f\right)\cdot dP.$$
  The mapping $f_{\#}$ from P(M,Σ) to $P(M^{\prime },\Sigma^{\prime })$ is continuous if these spaces are endowed with the total variation topology:
  $$\begin{array}{cc} ∥f_{\#}P-f_{\#}P^{\prime }{∥}_{TV} & \overset{\left(a\right)}{\leq }{∥P-P^{\prime }∥}_{TV}, \end{array}$$
  where (a) follows from Property 1 of [8].
- Probability measures on finite sets:
  We always endow finite sets with their finest σ-algebra, i.e., the power set. In this case, every probability measure is completely determined by its value on singletons, i.e., if *P* is a probability measure on a finite set X, then for every A⊂X, we have:
  $$P\left(A\right)=\sum_{x\in A}P\left(x\right).$$
  If X is a finite set, we denote the set of probability distributions on X as $\Delta_{X}$. Note that $\Delta_{X}$ is an (|X|−1)-dimensional simplex in $R^{X}$. We always endow $\Delta_{X}$ with the total variation distance and its induced topology. For every $p_{1},p_{2}\in \Delta_{X}$, we have:
  $$∥p_{1}-p_{2}{∥}_{TV}=\frac{1}{2}\sum_{x\in X}|p_{1}\left(x\right)-p_{2}\left(x\right)|=\frac{1}{2}{∥p_{1}-p_{2}∥}_{1}.$$
- Products of probability measures:
  We denote the product of two measurable spaces $\left(M_{1},\Sigma_{1}\right)$ and $\left(M_{2},\Sigma_{2}\right)$ as $\left(M_{1}\times M_{2},\Sigma_{1}⊗\Sigma_{2}\right)$. If $P_{1}\in P\left(M_{1},\Sigma_{1}\right)$ and $P_{2}\in P\left(M_{2},\Sigma_{2}\right)$, we denote the product of $P_{1}$ and $P_{2}$ as $P_{1}\times P_{2}$.
  If $P(M_{1},\Sigma_{1})$, $P(M_{2},\Sigma_{2})$ and $P(M_{1}\times M_{2},\Sigma_{1}⊗\Sigma_{2})$ are endowed with the total variation topology, the mapping $\left(P_{1},P_{2}\right)\to P_{1}\times P_{2}$ is a continuous mapping (see Appendix B).
- Borel sets and the support of a probability measure:
  Let (T,U) be a Hausdorff topological space. The Borel σ-algebra of (T,U) is the σ-algebra generated by U. We denote the Borel σ-algebra of (T,U) as B(T,U). If the topology U is known from the context, we simply write B(T) to denote the Borel σ-algebra. The sets in B(T) are called the Borel sets of *T*.

The support of a measure P∈P(T,B(T)) is the set of all points x∈T for which every neighborhood has a strictly positive measure:

supp(P)={x∈T:P(O)>0foreveryneighborhoodOofx}.

If *P* is a probability measure on a Polish space, then $P\left(T\backslash \mathrm{supp}(P)\right)=0$.

### 2.5. Random Mappings

Let *M* and $M^{\prime }$ be two arbitrary sets, and let $\Sigma^{\prime }$ be a σ-algebra on $M^{\prime }$. A random mapping from *M* to $\left(M^{\prime },\Sigma^{\prime }\right)$ is a mapping *R* from *M* to $P(M^{\prime },\Sigma^{\prime })$. For every x∈M, R(x) can be interpreted as the probability distribution of the random output given that the input is *x*.

Let Σ be a σ-algebra on *M*. We say that *R* is a measurable random mapping from (M,Σ) to $\left(M^{\prime },\Sigma^{\prime }\right)$ if the mapping $R_{B}:M\to R$ defined as $R_{B}\left(x\right)=\left(R\left(x\right)\right)\left(B\right)$ is measurable for every $B\in \Sigma^{\prime }$.

Note that this definition of measurability is consistent with the measurability of ordinary mappings: let *f* be a mapping from *M* to $M^{\prime }$, and let $D_{f}:M\to P\left(M^{\prime },\Sigma^{\prime }\right)$ be the random mapping defined as $D_{f}\left(x\right)=\delta_{f(x)}$ for every x∈M, where $\delta_{f(x)}\in P\left(M^{\prime },\Sigma^{\prime }\right)$ is a Dirac measure centered at f(x). We have:

$$\begin{array}{cc} D_{f}\;\mathrm{is}\;\mathrm{measurable}\;\; & \Leftrightarrow \;\;{\left(D_{f}\right)}_{B}\;\mathrm{is}\;\mathrm{measurable},\;\;\forall B\in \Sigma^{\prime }\\ & \Leftrightarrow \;\;{\left({\left(D_{f}\right)}_{B}\right)}^{-1}\left(B^{\prime }\right)\in \Sigma ,\;\;\forall B^{\prime }\in B\left(R\right),\;\forall B\in \Sigma^{\prime }\\ & \overset{\left(a\right)}{\Leftrightarrow }\;\;{\left({\left(D_{f}\right)}_{B}\right)}^{-1}\left(\left\{1\right\}\right)\in \Sigma ,\;\;\forall B\in \Sigma^{\prime }\\ & \overset{\left(b\right)}{\Leftrightarrow }\;\;f^{-1}\left(B\right)\in \Sigma ,\;\;\forall B\in \Sigma^{\prime }\\ & \Leftrightarrow \;\;f\;\mathrm{is}\;\mathrm{measurable}, \end{array}$$

where (a) and (b) follow from the fact that $\left({\left(D_{f}\right)}_{B}\right)\left(x\right)$ is either one or zero, depending on whether f(x)∈B or not.

Let *P* be a probability measure on (M,Σ), and let *R* be a measurable random mapping from (M,Σ) to $\left(M^{\prime },\Sigma^{\prime }\right)$. The push-forward probability measure of *P* by *R* is the probability measure $R_{\#}P$ on $\left(M^{\prime },\Sigma^{\prime }\right)$ defined as:

$$\left(R_{\#}P\right)\left(B\right)=\int_{M}R_{B}\cdot dP,\;\;\forall B\in \Sigma^{\prime }.$$

Note that this definition is consistent with the push-forward of ordinary mappings: if *f* and $D_{f}$ are as above, then for every $B\in \Sigma^{\prime }$, we have:

$$\left({\left(D_{f}\right)}_{\#}P\right)\left(B\right)=\int_{M}{\left(D_{f}\right)}_{B}\cdot dP=\int_{M}\left({𝟙}_{B}\circ f\right)\cdot dP=\int_{M^{\prime }}{𝟙}_{B}\cdot d\left(f_{\#}P\right)=\left(f_{\#}P\right)\left(B\right).$$

**Proposition** **1.** *Let R be a measurable random mapping from (M,Σ) to $\left(M^{\prime },\Sigma^{\prime }\right)$. If $g:M^{\prime }\to R^{+}\cup \left\{+\infty \right\}$ is a $\Sigma^{\prime }$-measurable mapping, then the mapping $x\to \int_{M^{\prime }}g\left(y\right)\cdot d\left(R\left(x\right)\right)\left(y\right)$ is a measurable mapping from (M,Σ) to $R^{+}\cup \left\{+\infty \right\}$. Moreover, for every P∈P(M,Σ), we have:*

$$\int_{M^{\prime }}g\cdot d\left(R_{\#}P\right)=\int_{M}\left(\int_{M^{\prime }}g\left(y\right)\cdot d\left(R\left(x\right)\right)\left(y\right)\right)dP\left(x\right).$$

**Proof.**  See Appendix C. ☐

**Corollary** **2.** *If $g:M^{\prime }\to R$ is bounded and $\Sigma^{\prime }$-measurable, then the mapping:*

$$x\to \int_{M^{\prime }}g\left(y\right)\cdot d\left(R\left(x\right)\right)\left(y\right)$$

*is bounded and* Σ*-measurable. Moreover, for every P∈P(M,Σ), we have:*

$$\int_{M^{\prime }}g\cdot d\left(R_{\#}P\right)=\int_{M}\left(\int_{M^{\prime }}g\left(y\right)\cdot d\left(R\left(x\right)\right)\left(y\right)\right)dP\left(x\right).$$

**Proof.**  Write $g=g^{+}-g^{-}$ (where $g^{+}=\max\left\{g,0\right\}$ and $g^{-}=\max\left\{-g,0\right\}$), and use the fact that every bounded measurable function is integrable over any probability distribution. ☐

**Lemma** **3.** *For every measurable random mapping R from (M,Σ) to $\left(M^{\prime },\Sigma^{\prime }\right)$, the push-forward mapping $R_{\#}$ is continuous from P(M,Σ) to $P(M^{\prime },\Sigma^{\prime })$ under the total variation topology.*

**Proof.**  See Appendix D. ☐

**Lemma** **4.** *Let U be a Polish (This assumption can be dropped. We assumed that U is Polish just to avoid working with Moore–Smith nets.) topology on M, and let $U^{\prime }$ be an arbitrary topology on $M^{\prime }$. Let R be a measurable random mapping from (M,B(M)) to $\left(M^{\prime },B\left(M^{\prime }\right)\right)$. Moreover, assume that R is a continuous mapping from (M,U) to $P(M^{\prime },B\left(M^{\prime }\right))$ when the latter space is endowed with the weak-∗ topology. Under these assumptions, the push-forward mapping $R_{\#}$ is continuous from P(M,B(M)) to $P(M^{\prime },B\left(M^{\prime }\right))$ under the weak-∗ topology.*

**Proof.**  See Appendix D. ☐

### 2.6. Meta-Probability Measures

Let X be a finite set. A meta-probability measure on X is a probability measure on the Borel sets of $\Delta_{X}$. It is called a meta-probability measure because it is a probability measure on the space of probability distributions on X.

We denote the set of meta-probability measures on X as MP(X). Clearly, $\mathrm{MP}\left(X\right)=P\left(\Delta_{X}\right)$.

A meta-probability measure MP on X is said to be balanced if it satisfies:

$$\int_{\Delta_{X}}p\cdot d\mathrm{MP}\left(p\right)=\pi_{X},$$

where $\pi_{X}$ is the uniform probability distribution on X.

We denote the set of all balanced meta-probability measures on X as ${\mathrm{MP}}_{b}\left(X\right)$. The set of all balanced and finitely-supported meta-probability measures on X is denoted as ${\mathrm{MP}}_{bf}\left(X\right)$.

The following lemma is useful to show the continuity of functions defined on MP(X).

**Lemma** **5.** *Let (S,V) be a compact topological space, and let $f:S\times \Delta_{X}\to R$ be a continuous function on $S\times \Delta_{X}$. The mapping F:S×MP(X)→R defined as:*

$$F\left(s,\mathrm{MP}\right)=\int_{\Delta_{X}}f\left(s,p\right)\cdot d\mathrm{MP}\left(p\right)$$

*is continuous, where MP(X) is endowed with the weak-∗ topology.*

**Proof.**  See Appendix E. ☐

Let *f* be a mapping from a finite set X to another finite set $X^{\prime }$. *f* induces a push-forward mapping $f_{\#}$ taking probability distributions in $\Delta_{X}$ to probability distributions in $\Delta_{X^{\prime }}$. $f_{\#}$ is continuous because $\Delta_{X}$ and $\Delta_{X^{\prime }}$ are endowed with the total variation distance. $f_{\#}$ in turn induces another push-forward mapping taking meta-probability measures in MP(X) to meta-probability measures in $\mathrm{MP}(X^{\prime })$. We denote this mapping as $f_{\#\#}$, and we call it the meta-push-forward mapping induced by *f*. Since $f_{\#}$ is a continuous mapping from $\Delta_{X}$ to $\Delta_{X^{\prime }}$, $f_{\#\#}$ is a continuous mapping from MP(X) to $\mathrm{MP}(X^{\prime })$ under both the weak-∗ and the total variation topologies.

Let $X_{1}$ and $X_{2}$ be two finite sets. Let $\mathrm{Mul}:\Delta_{X_{1}}\times \Delta_{X_{2}}\to \Delta_{X_{1}\times X_{2}}$ be defined as $\mathrm{Mul}\left(p_{1},p_{2}\right)=p_{1}\times p_{2}$. For every ${\mathrm{MP}}_{1}\in \mathrm{MP}\left(X_{1}\right)$ and ${\mathrm{MP}}_{2}\in \mathrm{MP}\left(X_{2}\right)$, we define the tensor product of ${\mathrm{MP}}_{1}$ and ${\mathrm{MP}}_{2}$ as ${\mathrm{MP}}_{1}⊗{\mathrm{MP}}_{2}={\mathrm{Mul}}_{\#}\left({\mathrm{MP}}_{1}\times {\mathrm{MP}}_{2}\right)\in \mathrm{MP}\left(X_{1}\times X_{2}\right)$.

Note that since $\Delta_{X_{1}}$, $\Delta_{X_{2}}$ and $\Delta_{X_{1}\times X_{2}}$ are endowed with the total variation topology, $\mathrm{Mul}\left(p_{1},p_{2}\right)=p_{1}\times p_{2}$ is a continuous mapping from $\Delta_{X_{1}}\times \Delta_{X_{2}}$ to $\Delta_{X_{1}\times X_{2}}$. Therefore, ${\mathrm{Mul}}_{\#}$ is a continuous mapping from $P(\Delta_{X_{1}}\times \Delta_{X_{2}})$ to $P\left(\Delta_{X_{1}\times X_{2}}\right)=\mathrm{MP}\left(X_{1}\times X_{2}\right)$ under both the weak-∗ and the total variation topologies. On the other hand, Appendix B and Appendix F imply that the mapping $\left({\mathrm{MP}}_{1},{\mathrm{MP}}_{2}\right)\to {\mathrm{MP}}_{1}\times {\mathrm{MP}}_{2}$ from $\mathrm{MP}\left(X_{1}\right)\times \mathrm{MP}\left(X_{2}\right)$ to $P(\Delta_{X_{1}}\times \Delta_{X_{2}})$ is continuous under both the weak-∗ and the total variation topologies. We conclude that the tensor product is continuous under both of these topologies.

## 3. The Space of Equivalent Channels

In this section, we summarize the main results of [6].

### 3.1. Space of Channels from X to Y

A discrete memoryless channel *W* is a three-tuple $W=(X,\;Y,\;p_{W})$ where X is a finite set that is called the input alphabet of *W*, Y is a finite set that is called the output alphabet of *W* and $p_{W}:X\times Y\to \left[0,1\right]$ is a function satisfying $\forall x\in X,\;\sum_{y\in Y}p_{W}\left(x,y\right)=1$.

For every (x,y)∈X×Y, we denote $p_{W}\left(x,y\right)$ as W(y|x), which we interpret as the conditional probability of receiving *y* at the output, given that *x* is the input.

Let ${\mathrm{DMC}}_{X,Y}$ be the set of all channels having X as the input alphabet and Y as the output alphabet.

For every $W,W^{\prime }\in {\mathrm{DMC}}_{X,Y}$, define the distance between *W* and $W^{\prime }$ as follows:

$$d_{X,Y}\left(W,W^{\prime }\right)=\frac{1}{2}\max_{x\in X}\sum_{y\in Y}\left|W^{\prime }\left(y|x\right)-W\left(y|x\right)\right|.$$

We always endow ${\mathrm{DMC}}_{X,Y}$ with the metric distance $d_{X,Y}$. This metric makes ${\mathrm{DMC}}_{X,Y}$ a compact path-connected metric space. The metric topology on ${\mathrm{DMC}}_{X,Y}$ that is induced by $d_{X,Y}$ is denoted as $T_{X,Y}$.

### 3.2. Equivalence between Channels

Let $W\in {\mathrm{DMC}}_{X,Y}$ and $W^{\prime }\in {\mathrm{DMC}}_{X,Z}$ be two channels having the same input alphabet. We say that $W^{\prime }$ is degraded from *W* if there exists a channel $V\in {\mathrm{DMC}}_{Y,Z}$ such that:

$$W^{\prime }\left(z|x\right)=\sum_{y\in Y}V\left(z|y\right)W\left(y|x\right).$$

*W* and $W^{\prime }$ are said to be equivalent if each one is degraded from the other.

Let $\Delta_{X}$ and $\Delta_{Y}$ be the space of probability distributions on X and Y, respectively. Define $P_{W}^{o}\in \Delta_{Y}$ as $P_{W}^{o}\left(y\right)=\frac{1}{\left|X\right|}\sum_{x\in X}W\left(y|x\right)$ for every y∈Y. The image of *W* is the set of output-symbols y∈Y having strictly positive probabilities:

Im(W)={y∈Y:PWo(y)>0}.

For every y∈Im(W), define $W_{y}^{-1}\in \Delta_{X}$ as follows:

$$W_{y}^{-1}\left(x\right)=\frac{W(y|x)}{\left|X\right|P_{W}^{o}\left(y\right)},\;\;\forall x\in X.$$

For every (x,y)∈X×Im(W), we have $W\left(y|x\right)=\left|X\right|P_{W}^{o}\left(y\right)W_{y}^{-1}\left(x\right)$. On the other hand, if x∈X and y∈Y\Im(W), we have W(y|x)=0. This shows that $P_{W}^{o}$ and the collection {Wy−1}y∈Im(W) uniquely determine *W*.

The Blackwell measure (denoted ${\mathrm{MP}}_{W}$) of *W* is a meta-probability measure on X defined as:

MPW=∑y∈Im(W)PWo(y)δWy−1,

where $\delta_{W_{y}^{-1}}$ is a Dirac measure centered at $W_{y}^{-1}$. In an earlier version of this work, I called ${\mathrm{MP}}_{W}$ the posterior meta-probability distribution of *W*. Maxim Raginsky thankfully brought to my attention the fact that ${\mathrm{MP}}_{W}$ is called the Blackwell measure.

It is known that a meta-probability measure MP on X is the Blackwell measure of some discrete memoryless channels (DMC) with input alphabet X if and only if it is balanced and finitely supported [9].

It is also known that two channels $W\in {\mathrm{DMC}}_{X,Y}$ and $W^{\prime }\in {\mathrm{DMC}}_{X,Z}$ are equivalent if and only if ${\mathrm{MP}}_{W}={\mathrm{MP}}_{W^{\prime }}$ [9].

### 3.3. Space of Equivalent Channels from X to Y

Let X and Y be two finite sets. Define the equivalence relation $R_{X,Y}^{\left(o\right)}$ on ${\mathrm{DMC}}_{X,Y}$ as follows:

$$\forall W,W^{\prime }\in {\mathrm{DMC}}_{X,Y},\;\;WR_{X,Y}^{\left(o\right)}W^{\prime }\;\;\Leftrightarrow \;\;W\;\mathrm{is}\;\mathrm{equivalent}\;\mathrm{to}\;W^{\prime }.$$

The space of equivalent channels with input alphabet X and output alphabet Y is the quotient of ${\mathrm{DMC}}_{X,Y}$ by the equivalence relation:

$${\mathrm{DMC}}_{X,Y}^{\left(o\right)}={\mathrm{DMC}}_{X,Y}/R_{X,Y}^{\left(o\right)}.$$

Quotient topology: 

We define the topology $T_{X,Y}^{\left(o\right)}$ on ${\mathrm{DMC}}_{X,Y}^{\left(o\right)}$ as the quotient topology $T_{X,Y}/R_{X,Y}^{\left(o\right)}$. We always associate ${\mathrm{DMC}}_{X,Y}^{\left(o\right)}$ with the quotient topology $T_{X,Y}^{\left(o\right)}$.

We have shown in [6] that ${\mathrm{DMC}}_{X,Y}^{\left(o\right)}$ is a compact, path-connected and metrizable space.

If $Y_{1}$ and $Y_{2}$ are two finite sets of the same size, there exists a canonical homeomorphism between ${\mathrm{DMC}}_{X,Y_{1}}^{\left(o\right)}$ and ${\mathrm{DMC}}_{X,Y_{2}}^{\left(o\right)}$ [6]. This allows us to identify ${\mathrm{DMC}}_{X,Y}^{\left(o\right)}$ with ${\mathrm{DMC}}_{X,[n]}^{\left(o\right)}$, where n=|Y| and [n]={1,…,n}.

Moreover, for every 1≤n≤m, there exists a canonical subspace of ${\mathrm{DMC}}_{X,[m]}^{\left(o\right)}$ that is homeomorphic to ${\mathrm{DMC}}_{X,[n]}^{\left(o\right)}$ [6]. Therefore, we can consider ${\mathrm{DMC}}_{X,[n]}^{\left(o\right)}$ as a compact subspace of ${\mathrm{DMC}}_{X,[m]}^{\left(o\right)}$.

Noisiness metric: 

For every m≥1, let $\Delta_{[m]\times X}$ be the space of probability distributions on [m]×X. Let Y be a finite set, and let $W\in {\mathrm{DMC}}_{X,Y}$. For every $p\in \Delta_{[m]\times X}$, define $P_{c}\left(p,W\right)$ as follows:

$$P_{c}\left(p,W\right)=\underset{D\in {\mathrm{DMC}}_{Y,[m]}}{\sup}\sum_{\begin{array}{c} u\in [m],\\ x\in X,\\ y\in Y \end{array}}p\left(u,x\right)W\left(y|x\right)D\left(u|y\right).$$

The quantity $P_{c}\left(p,W\right)$ depends only on the $R_{X,Y}^{\left(o\right)}$-equivalence class of *W* (see [6]). Therefore, if $\hat{W}\in {\mathrm{DMC}}_{X,Y}^{\left(o\right)}$, we can define $P_{c}\left(p,\hat{W}\right):=P_{c}\left(p,W^{\prime }\right)$ for any $W^{\prime }\in \hat{W}$.

Define the noisiness distance $d_{X,Y}^{\left(o\right)}:{\mathrm{DMC}}_{X,Y}^{\left(o\right)}\times {\mathrm{DMC}}_{X,Y}^{\left(o\right)}\to R^{+}$ as follows:

$$d_{X,Y}^{\left(o\right)}\left({\hat{W}}_{1},{\hat{W}}_{2}\right)=\underset{\begin{array}{c} m\geq 1,\\ p\in \Delta_{[m]\times X} \end{array}}{\sup}\left|P_{c}\left(p,{\hat{W}}_{1}\right)-P_{c}\left(p,{\hat{W}}_{2}\right)\right|.$$

We have shown in [6] that $\left({\mathrm{DMC}}_{X,Y}^{\left(o\right)},T_{X,Y}^{\left(o\right)}\right)$ is topologically equivalent to $\left({\mathrm{DMC}}_{X,Y}^{\left(o\right)},d_{X,Y}^{\left(o\right)}\right)$.

### 3.4. Space of Equivalent Channels with Input Alphabet X

The space of channels with input alphabet X is defined as:

$${\mathrm{DMC}}_{X,*}=\underset{n\geq 1}{∐}{\mathrm{DMC}}_{X,[n]}.$$

We define the equivalence relation $R_{X,*}^{\left(o\right)}$ on ${\mathrm{DMC}}_{X,*}$ as follows:

$$\forall W,W^{\prime }\in {\mathrm{DMC}}_{X,*},\;\;WR_{X,*}^{\left(o\right)}W^{\prime }\;\;\Leftrightarrow \;\;W\;\mathrm{is}\;\mathrm{equivalent}\;\mathrm{to}\;W^{\prime }.$$

The space of equivalent channels with input alphabet X is the quotient of ${\mathrm{DMC}}_{X,*}$ by the equivalence relation:

$${\mathrm{DMC}}_{X,*}^{\left(o\right)}={\mathrm{DMC}}_{X,*}/R_{X,*}^{\left(o\right)}.$$

For every n≥1 and every $W\in {\mathrm{DMC}}_{X,[n]}$, we identify the $R_{X,[n]}^{\left(o\right)}$-equivalence class of *W* with the $R_{X,*}^{\left(o\right)}$-equivalence class of it. This allows us to consider ${\mathrm{DMC}}_{X,[n]}^{\left(o\right)}$ as a subspace of ${\mathrm{DMC}}_{X,*}^{\left(o\right)}$. Moreover,

$${\mathrm{DMC}}_{X,*}^{\left(o\right)}=\underset{n\geq 1}{⋃}{\mathrm{DMC}}_{X,[n]}^{\left(o\right)}.$$

Since any two equivalent channels have the same Blackwell measure, we can define the Blackwell measure of $\hat{W}\in {\mathrm{DMC}}_{X,*}^{\left(o\right)}$ as ${\mathrm{MP}}_{\hat{W}}={\mathrm{MP}}_{W^{\prime }}$ for any $W^{\prime }\in \hat{W}$. The rank of $\hat{W}\in {\mathrm{DMC}}_{X,*}^{\left(o\right)}$ is the size of the support of its Blackwell measure:

$$\mathrm{rank}\left(\hat{W}\right)=\left|\mathrm{supp}\left({\mathrm{MP}}_{\hat{W}}\right)\right|.$$

We have:

$${\mathrm{DMC}}_{X,[n]}^{\left(o\right)}=\left\{\hat{W}\in {\mathrm{DMC}}_{X,*}^{\left(o\right)}:\;\mathrm{rank}\left(\hat{W}\right)\leq n\right\}.$$

A topology T on ${\mathrm{DMC}}_{X,*}^{\left(o\right)}$ is said to be natural if and only if it induces the quotient topology $T_{X,[n]}^{\left(o\right)}$ on ${\mathrm{DMC}}_{X,[n]}^{\left(o\right)}$ for every n≥1.

Every natural topology is σ-compact, separable and path-connected [6]. On the other hand, if |X|≥2, a Hausdorff natural topology is not Baire, and it is not locally compact anywhere [6]. This implies that no natural topology can be completely metrized if |X|≥2.

Strong topology on ${\mathrm{DMC}}_{X,*}^{\left(o\right)}$: 

We associate ${\mathrm{DMC}}_{X,*}$ with the disjoint union topology $T_{s,X,*}:=\underset{n\geq 1}{⨁}T_{X,[n]}$. The space $\left({\mathrm{DMC}}_{X,*},T_{s,X,*}\right)$ is disconnected, metrizable and σ-compact [6].

The strong topology $T_{s,X,*}^{\left(o\right)}$ on ${\mathrm{DMC}}_{X,*}^{\left(o\right)}$ is the quotient of $T_{s,X,*}$ by $R_{X,*}^{\left(o\right)}$:

$$T_{s,X,*}^{\left(o\right)}=T_{s,X,*}/R_{X,*}^{\left(o\right)}.$$

We call open and closed sets in $\left({\mathrm{DMC}}_{X,*}^{\left(o\right)},T_{s,X,*}^{\left(o\right)}\right)$ as strongly-open and strongly-closed sets, respectively. If *A* is a subset of ${\mathrm{DMC}}_{X,*}^{\left(o\right)}$, then *A* is strongly open if and only if $A\cap {\mathrm{DMC}}_{X,[n]}^{\left(o\right)}$ is open in ${\mathrm{DMC}}_{X,[n]}^{\left(o\right)}$ for every n≥1. Similarly, *A* is strongly closed if and only if $A\cap {\mathrm{DMC}}_{X,[n]}^{\left(o\right)}$ is closed in ${\mathrm{DMC}}_{X,[n]}^{\left(o\right)}$ for every n≥1.

We have shown in [6] that $T_{s,X,*}^{\left(o\right)}$ is the finest natural topology. The strong topology is sequential, compactly generated and $T_{4}$ [6]. On the other hand, if |X|≥2, the strong topology is not first-countable anywhere [6]; hence, it is not metrizable.

Noisiness metric: 

Define the noisiness metric on ${\mathrm{DMC}}_{X,*}^{\left(o\right)}$ as follows:

$$d_{X,*}^{\left(o\right)}\left(\hat{W},{\hat{W}}^{\prime }\right):=d_{X,[n]}^{\left(o\right)}\left(\hat{W},{\hat{W}}^{\prime }\right)\;\mathrm{where}\;n\geq 1\;\mathrm{satisfies}\;\hat{W},{\hat{W}}^{\prime }\in {\mathrm{DMC}}_{X,[n]}^{\left(o\right)}.$$

$d_{X,*}^{\left(o\right)}\left(\hat{W},{\hat{W}}^{\prime }\right)$ is well-defined because $d_{X,[n]}^{\left(o\right)}\left(\hat{W},{\hat{W}}^{\prime }\right)$ does not depend on n≥1 as long as $\hat{W},{\hat{W}}^{\prime }\in {\mathrm{DMC}}_{X,[n]}^{\left(o\right)}$. We can also express $d_{X,*}^{\left(o\right)}$ as follows:

$$d_{X,*}^{\left(o\right)}\left(\hat{W},{\hat{W}}^{\prime }\right)=\underset{\begin{array}{c} m\geq 1,\\ p\in \Delta_{[m]\times X} \end{array}}{\sup}\left|P_{c}\left(p,\hat{W}\right)-P_{c}\left(p,{\hat{W}}^{\prime }\right)\right|.$$

The metric topology on ${\mathrm{DMC}}_{X,*}^{\left(o\right)}$ that is induced by $d_{X,*}^{\left(o\right)}$ is called the noisiness topology on ${\mathrm{DMC}}_{X,*}^{\left(o\right)}$, and it is denoted as $T_{X,*}^{\left(o\right)}$. We have shown in [6] that $T_{X,*}^{\left(o\right)}$ is a natural topology that is strictly coarser than $T_{s,X,*}^{\left(o\right)}$.

Topologies from Blackwell measures: 

The mapping $\hat{W}\to {\mathrm{MP}}_{\hat{W}}$ is a bijection from ${\mathrm{DMC}}_{X,*}^{\left(o\right)}$ to ${\mathrm{MP}}_{bf}\left(X\right)$. We call this mapping the canonical bijection from ${\mathrm{DMC}}_{X,*}^{\left(o\right)}$ to ${\mathrm{MP}}_{bf}\left(X\right)$.

Since $\Delta_{X}$ is a metric space, there are many standard ways to construct topologies on MP(X). If we choose any of these standard topologies on MP(X) and then relativize it to the subspace ${\mathrm{MP}}_{bf}\left(X\right)$, we can construct topologies on ${\mathrm{DMC}}_{X,*}^{\left(o\right)}$ through the canonical bijection.

In [6], we studied the weak-∗ and the total variation topologies. We showed that the weak-∗ topology is exactly the same as the noisiness topology.

The total-variation metric distance $d_{TV,X,*}^{\left(o\right)}$ on ${\mathrm{DMC}}_{X,*}^{\left(o\right)}$ is defined as:

$$d_{TV,X,*}^{\left(o\right)}\left(\hat{W},{\hat{W}}^{\prime }\right)={∥{\mathrm{MP}}_{\hat{W}}-{\mathrm{MP}}_{{\hat{W}}^{\prime }}∥}_{TV}.$$

The total-variation topology $T_{TV,X,*}^{\left(o\right)}$ is the metric topology that is induced by $d_{TV,X,*}^{\left(o\right)}$ on ${\mathrm{DMC}}_{X,*}^{\left(o\right)}$. We proved in [6] that if |X|≥2, we have:

- $T_{TV,X,*}^{\left(o\right)}$ is not natural, nor Baire, hence it is not completely metrizable.
- $T_{TV,X,*}^{\left(o\right)}$ is not locally compact anywhere.

## 4. Channel Parameters and Operations

### 4.1. Useful Parameters

Let $\Delta_{X}$ be the space of probability distributions on X. For every $p\in \Delta_{X}$ and every $W\in {\mathrm{DMC}}_{X,Y}$, define I(p,W) as the mutual information I(X;Y), where *X* is distributed as *p* and *Y* is the output of *W* when *X* is the input. The mutual information is computed using the natural logarithm. The capacity of *W* is defined as $C\left(W\right)=\underset{p\in \Delta_{X}}{\sup}I\left(p,W\right)$.

For every $p\in \Delta_{X}$, the error probability of the MAP decoder of *W* under prior *p* is defined as:

$$P_{e}\left(p,W\right)=1-\sum_{y\in Y}\max_{x\in X}\left\{p\left(x\right)W\left(y|x\right)\right\}.$$

Clearly, $0\leq P_{e}\left(p,W\right)\leq 1$.

For every $W\in {\mathrm{DMC}}_{X,Y}$, define the Bhattacharyya parameter of *W* as:

$$Z\left(W\right)=\left\{\begin{array}{cc} \frac{1}{\left|X|\cdot (|X|-1\right)}\sum_{\begin{array}{c} x_{1},x_{2}\in X,\\ x_{1}\neq x_{2} \end{array}}\sum_{y\in Y}\sqrt{W\left(y|x_{1}\right)W\left(y|x_{2}\right)},\; & \mathrm{if}\;|X|\geq 2\\ 0\; & \mathrm{if}\;|X|=1. \end{array}\right.$$

It is easy to see that 0≤Z(W)≤1.

It was shown in [10,11] that $\frac{1}{4}Z{\left(W\right)}^{2}\leq P_{e}\left(\pi_{X},W\right)\leq \left(|X|-1\right)Z\left(W\right)$, where $\pi_{X}$ is the uniform distribution on X.

An (n,M)-code C on the alphabet X is a subset of $X^{n}$ such that |C|=M. The integer *n* is the block length of C, and *M* is the size of the code. The rate of C is $\frac{1}{n}\log M$, and it is measured in nats. The error probability of the ML decoder for the code C when it is used for a channel $W\in {\mathrm{DMC}}_{X,Y}$ is given by:

$$P_{e,C}\left(W\right)=1-\frac{1}{\left|C\right|}\sum_{y_{1}^{n}\in Y^{n}}\max_{x_{1}^{n}\in C}\left\{\prod_{i=1}^{n}W\left(y_{i}|x_{i}\right)\right\}.$$

The optimal error probability of (n,M)-codes for a channel *W* is given by:

$$P_{e,n,M}\left(W\right)=\min_{\begin{array}{c} C\subset X^{n},\\ |C|=M \end{array}}P_{e,C}\left(W\right).$$

The following proposition shows that all the above parameters are continuous:

**Proposition** **2.** *We have:*

- *$I:\Delta_{X}\times {\mathrm{DMC}}_{X,Y}\to R^{+}$ is continuous, concave in p and convex in W.*
- *$C:{\mathrm{DMC}}_{X,Y}\to R^{+}$ is continuous and convex.*
- *$P_{e}:\Delta_{X}\times {\mathrm{DMC}}_{X,Y}\to \left[0,1\right]$ is continuous, concave in p and concave in W.*
- *$Z:{\mathrm{DMC}}_{X,Y}\to \left[0,1\right]$ is continuous.*
- *For every code C on X, $P_{e,C}:{\mathrm{DMC}}_{X,Y}\to \left[0,1\right]$ is continuous.*
- *For every n>0 and every $1\leq M\leq {\left|X\right|}^{n}$, the mapping $P_{e,n,M}:{\mathrm{DMC}}_{X,Y}\to \left[0,1\right]$ is continuous.*

**Proof.**  These facts are well known, especially the continuity of *I*, its concavity in *p* and its convexity in *W* [12]. Since *C* is the supremum of a family of mappings that are convex in *W*, it is also convex in *W*. For a proof of the continuity of *C*, see Appendix G. The continuity of *Z*, $P_{e}$ and $P_{e,C}$ follows immediately from their definitions. Moreover, since $P_{e,n,M}$ is the minimum of a finite number of continuous mappings, it is continuous. The concavity of $P_{e}$ in *p* and in *W* can also be easily seen from the definition. ☐

### 4.2. Channel Operations

If $W\in {\mathrm{DMC}}_{X,Y}$ and $V\in {\mathrm{DMC}}_{Y,Z}$, we define the composition $V\circ W\in {\mathrm{DMC}}_{X,Z}$ of *W* and *V* as follows:

$$\left(V\circ W\right)\left(z|x\right)=\sum_{y\in Y}V\left(z|y\right)W\left(y|x\right).\;\;\forall x\in X,\;\forall z\in Z.$$

For every function f:X→Y, define the deterministic channel $D_{f}\in {\mathrm{DMC}}_{X,Y}$ as follows:

$$D_{f}\left(y|x\right)=\left\{\begin{array}{cc} 1\; & \mathrm{if}\;y=f(x),\\ 0\; & \mathrm{otherwise}. \end{array}\right.$$

It is easy to see that if f:X→Y and g:Y→Z, then $D_{g}\circ D_{f}=D_{g\circ f}$.

For every two channels $W_{1}\in {\mathrm{DMC}}_{X_{1},Y_{1}}$ and $W_{2}\in {\mathrm{DMC}}_{X_{2},Y_{2}}$, define the channel sum $W_{1}⊕W_{2}\in {\mathrm{DMC}}_{X_{1}∐X_{2},Y_{1}∐Y_{2}}$ of $W_{1}$ and $W_{2}$ as:

$$\left(W_{1}⊕W_{2}\right)\left(y,i|x,j\right)=\left\{\begin{array}{cc} W_{i}\left(y|x\right)\; & \mathrm{if}\;i=j,\\ 0 & \mathrm{otherwise}. \end{array}\right.$$

$W_{1}⊕W_{2}$ arises when the transmitter has two channels $W_{1}$ and $W_{2}$ at its disposal, and it can use exactly one of them at each channel use. It is an easy exercise to check that $e^{C(W_{1}⊕W_{2})}=e^{C(W_{1})}+e^{C(W_{2})}$ (remember that we compute the mutual information using the natural logarithm).

We define the channel product $W_{1}⊗W_{2}\in {\mathrm{DMC}}_{X_{1}\times X_{2},Y_{1}\times Y_{2}}$ of $W_{1}$ and $W_{2}$ as:

$$\left(W_{1}⊗W_{2}\right)\left(y_{1},y_{2}|x_{1},x_{2}\right)=W_{1}\left(y_{1}|x_{1}\right)W_{2}\left(y_{2}|x_{2}\right).$$

$W_{1}⊗W_{2}$ arises when the transmitter has two channels $W_{1}$ and $W_{2}$ at its disposal, and it uses both of them at each channel use. It is an easy exercise to check that $C\left(W_{1}⊗W_{2}\right)=C\left(W_{1}\right)+C\left(W_{2}\right)$, or equivalently $e^{C(W_{1}⊗W_{2})}=e^{C(W_{1})}\cdot e^{C(W_{2})}$. Channel sums and products were first introduced by Shannon in [13].

For every $W_{1}\in {\mathrm{DMC}}_{X,Y_{1}}$, $W_{2}\in {\mathrm{DMC}}_{X,Y_{2}}$ and every 0≤α≤1, we define the α-interpolation $\left[\alpha W_{1},\left(1-\alpha \right)W_{2}\right]\in {\mathrm{DMC}}_{X,Y_{1}∐Y_{2}}$ between $W_{1}$ and $W_{2}$ as:

$$\left[\alpha W_{1},\left(1-\alpha \right)W_{2}\right]\left(y,i|x\right)=\left\{\begin{array}{cc} \alpha W_{1}\left(y|x\right)\; & \mathrm{if}\;i=1,\\ \left(1-\alpha \right)W_{2}\left(y|x\right)\; & \mathrm{if}\;i=2. \end{array}\right.$$

Channel interpolation arises when a channel behaves as $W_{1}$ with probability α and as $W_{2}$ with probability 1−α. The transmitter has no control on which behavior the channel chooses, but on the other hand, the receiver knows which one was chosen. Channel interpolations were used in [14] to construct interpolations between polar codes and Reed–Muller codes.

Now, fix a binary operation ∗ on X. For every $W\in {\mathrm{DMC}}_{X,Y}$, define $W^{-}\in {\mathrm{DMC}}_{X,Y^{2}}$ and $W^{+}\in {\mathrm{DMC}}_{X,Y^{2}\times X}$ as:

$$W^{-}\left(y_{1},y_{2}|u_{1}\right)=\frac{1}{\left|X\right|}\sum_{u_{2}\in X}W\left(y_{1}|u_{1}*u_{2}\right)W\left(y_{2}|u_{2}\right),$$

and:

$$W^{+}\left(y_{1},y_{2},u_{1}|u_{2}\right)=\frac{1}{\left|X\right|}W\left(y_{1}|u_{1}*u_{2}\right)W\left(y_{2}|u_{2}\right).$$

These operations generalize Arıkan’s polarization transformations [15].

**Proposition** **3.** *We have:*

- *The mapping (W,V)→V∘W from ${\mathrm{DMC}}_{X,Y}\times {\mathrm{DMC}}_{Y,Z}$ to ${\mathrm{DMC}}_{X,Z}$ is continuous.*
- *The mapping $\left(W_{1},W_{2}\right)\to W_{1}⊕W_{2}$ from ${\mathrm{DMC}}_{X_{1},Y_{1}}\times {\mathrm{DMC}}_{X_{2},Y_{2}}$ to the space ${\mathrm{DMC}}_{X_{1}∐X_{2},Y_{1}∐Y_{2}}$ is continuous.*
- *The mapping $\left(W_{1},W_{2}\right)\to W_{1}⊗W_{2}$ from ${\mathrm{DMC}}_{X_{1},Y_{1}}\times {\mathrm{DMC}}_{X_{2},Y_{2}}$ to ${\mathrm{DMC}}_{X_{1}\times X_{2},Y_{1}\times Y_{2}}$ is continuous.*
- *The mapping $\left(W_{1},W_{2},\alpha \right)\to \left[\alpha W_{1},\left(1-\alpha \right)W_{2}\right]$ from ${\mathrm{DMC}}_{X,Y_{1}}\times {\mathrm{DMC}}_{X,Y_{2}}\times \left[0,1\right]$ to ${\mathrm{DMC}}_{X,Y_{1}∐Y_{2}}$ is continuous.*
- *For any binary operation ∗ on X, the mapping $W\to W^{-}$ from ${\mathrm{DMC}}_{X,Y}$ to ${\mathrm{DMC}}_{X,Y^{2}}$ is continuous.*
- *For any binary operation ∗ on X, the mapping $W\to W^{+}$ from ${\mathrm{DMC}}_{X,Y}$ to ${\mathrm{DMC}}_{X,Y^{2}\times X}$ is continuous.*

**Proof.**  The continuity immediately follows from the definitions. ☐

## 5. Continuity on ${\mathrm{DMC}}_{X,Y}^{\left(o\right)}$

It is well known that the parameters defined in Section 4.1 depend only on the $R_{X,Y}^{\left(o\right)}$-equivalence class of *W*. Therefore, we can define those parameters for any $\hat{W}\in {\mathrm{DMC}}_{X,Y}^{\left(o\right)}$ through the transcendent mapping (defined in Lemma 2). The following proposition shows that those parameters are continuous on ${\mathrm{DMC}}_{X,Y}^{\left(o\right)}$:

**Proposition** **4.** *We have:*

- *$I:\Delta_{X}\times {\mathrm{DMC}}_{X,Y}^{\left(o\right)}\to R^{+}$ is continuous and concave in p.*
- *$C:{\mathrm{DMC}}_{X,Y}^{\left(o\right)}\to R^{+}$ is continuous.*
- *$P_{e}:\Delta_{X}\times {\mathrm{DMC}}_{X,Y}^{\left(o\right)}\to \left[0,1\right]$ is continuous and concave in p.*
- *$Z:{\mathrm{DMC}}_{X,Y}^{\left(o\right)}\to \left[0,1\right]$ is continuous.*
- *For every code C on X, $P_{e,C}:{\mathrm{DMC}}_{X,Y}^{\left(o\right)}\to \left[0,1\right]$ is continuous.*
- *For every n>0 and every $1\leq M\leq {\left|X\right|}^{n}$, the mapping $P_{e,n,M}:{\mathrm{DMC}}_{X,Y}^{\left(o\right)}\to \left[0,1\right]$ is continuous.*

**Proof.**  Since the corresponding parameters are continuous on ${\mathrm{DMC}}_{X,Y}$ (Proposition 2), Lemma 2 implies that they are continuous on ${\mathrm{DMC}}_{X,Y}^{\left(o\right)}$. The only cases that need a special treatment are those of *I* and *Z*. We will only prove the continuity of *I* since the proof of continuity of *Z* is similar.

Define the relation *R* on $\Delta_{X}\times {\mathrm{DMC}}_{X,Y}$ as:

$$\left(p_{1},W_{1}\right)R\left(p_{2},W_{2}\right)\;\;\Leftrightarrow \;\;p_{1}=p_{2}\;\mathrm{and}\;W_{1}R_{X,Y}^{\left(o\right)}W_{2}.$$

It is easy to see that I(p,W) depends only on the *R*-equivalence class of (p,W). Since *I* is continuous on $\Delta_{X}\times {\mathrm{DMC}}_{X,Y}$, Lemma 2 implies that the transcendent mapping of *I* is continuous on $(\Delta_{X}\times {\mathrm{DMC}}_{X,Y})/R$. On the other hand, since $\Delta_{X}$ is locally compact, Theorem 1 implies that $(\Delta_{X}\times {\mathrm{DMC}}_{X,Y})/R$ can be identified with $\Delta_{X}\times \left({\mathrm{DMC}}_{X,Y}/R_{X,Y}^{\left(o\right)}\right)=\Delta_{X}\times {\mathrm{DMC}}_{X,Y}^{\left(o\right)}$, and the two spaces have the same topology. Therefore, *I* is continuous on $\Delta_{X}\times {\mathrm{DMC}}_{X,Y}^{\left(o\right)}$. ☐

With the exception of channel composition, all the channel operations that were defined in Section 4.2 can also be “quotiented”. We just need to realize that the equivalence class of the resulting channel depends only on the equivalence classes of the channels that were used in the operation. Let us illustrate this in the case of channel sums:

Let $W_{1},W_{1}^{\prime }\in {\mathrm{DMC}}_{X_{1},Y_{1}}$ and $W_{2},W_{2}^{\prime }\in {\mathrm{DMC}}_{X_{2},Y_{2}}$ and assume that $W_{1}$ is degraded from $W_{1}^{\prime }$ and $W_{2}$ is degraded from $W_{2}^{\prime }$. There exists $V_{1}\in {\mathrm{DMC}}_{Y_{1},Y_{1}}$ and $V_{2}\in {\mathrm{DMC}}_{Y_{2},Y_{2}}$ such that $W_{1}=V_{1}\circ W_{1}^{\prime }$ and $W_{2}=V_{2}\circ W_{2}^{\prime }$. It is easy to see that $W_{1}⊕W_{2}=\left(V_{1}⊕V_{2}\right)\circ \left(W_{1}^{\prime }⊕W_{2}^{\prime }\right)$, which shows that $W_{1}⊕W_{2}$ is degraded from $W_{1}^{\prime }⊕W_{2}^{\prime }$. This was proven by Shannon in [16].

Therefore, if $W_{1}$ is equivalent to $W_{1}^{\prime }$ and $W_{2}$ is equivalent to $W_{2}^{\prime }$, then $W_{1}⊕W_{2}$ is equivalent to $W_{1}^{\prime }⊕W_{2}^{\prime }$. This allows us to define the channel sum for every ${\hat{W}}_{1}\in {\mathrm{DMC}}_{X_{1},Y_{1}}^{\left(o\right)}$ and every ${\bar{W}}_{2}\in {\mathrm{DMC}}_{X_{2},Y_{2}}^{\left(o\right)}$ as ${\hat{W}}_{1}⊕{\bar{W}}_{2}=\tilde{W_{1}^{\prime }⊕W_{2}^{\prime }}\in {\mathrm{DMC}}_{X_{1}∐X_{2},Y_{1}∐Y_{2}}^{\left(o\right)}$ for any $W_{1}^{\prime }\in {\hat{W}}_{1}$ and any $W_{2}^{\prime }\in {\bar{W}}_{2}$, where $\tilde{W_{1}^{\prime }⊕W_{2}^{\prime }}$ is the $R_{X_{1}∐X_{2},Y_{1}∐Y_{2}}^{\left(o\right)}$-equivalence class of $W_{1}^{\prime }⊕W_{2}^{\prime }$.

With the exception of channel composition, we can “quotient” all the channel operations of Section 4.2 in a similar fashion. Moreover, we can show that they are continuous:

**Proposition** **5.** *We have:*

- *The mapping $\left({\hat{W}}_{1},{\bar{W}}_{2}\right)\to {\hat{W}}_{1}⊕{\bar{W}}_{2}$ from ${\mathrm{DMC}}_{X_{1},Y_{1}}^{\left(o\right)}\times {\mathrm{DMC}}_{X_{2},Y_{2}}^{\left(o\right)}$ to ${\mathrm{DMC}}_{X_{1}∐X_{2},Y_{1}∐Y_{2}}^{\left(o\right)}$ is continuous.*
- *The mapping $\left({\hat{W}}_{1},{\bar{W}}_{2}\right)\to {\hat{W}}_{1}⊗{\bar{W}}_{2}$ from ${\mathrm{DMC}}_{X_{1},Y_{1}}^{\left(o\right)}\times {\mathrm{DMC}}_{X_{2},Y_{2}}^{\left(o\right)}$ to ${\mathrm{DMC}}_{X_{1}\times X_{2},Y_{1}\times Y_{2}}^{\left(o\right)}$ is continuous.*
- *The mapping $\left({\hat{W}}_{1},{\bar{W}}_{2},\alpha \right)\to \left[\alpha {\hat{W}}_{1},\left(1-\alpha \right){\bar{W}}_{2}\right]$ from ${\mathrm{DMC}}_{X,Y_{1}}^{\left(o\right)}\times {\mathrm{DMC}}_{X,Y_{2}}^{\left(o\right)}\times \left[0,1\right]$ to ${\mathrm{DMC}}_{X,Y_{1}∐Y_{2}}^{\left(o\right)}$ is continuous.*
- *For any binary operation ∗ on X, the mapping $\hat{W}\to {\hat{W}}^{-}$ from ${\mathrm{DMC}}_{X,Y}^{\left(o\right)}$ to ${\mathrm{DMC}}_{X,Y^{2}}^{\left(o\right)}$ is continuous.*
- *For any binary operation ∗ on X, the mapping $\hat{W}\to {\hat{W}}^{+}$ from ${\mathrm{DMC}}_{X,Y}^{\left(o\right)}$ to ${\mathrm{DMC}}_{X,Y^{2}\times X}^{\left(o\right)}$ is continuous.*

**Proof.**  We only prove the continuity of the channel sum because the proof of continuity of the other operations is similar.

Let $\mathrm{Proj}:{\mathrm{DMC}}_{X_{1}∐X_{2},Y_{1}∐Y_{2}}\to {\mathrm{DMC}}_{X_{1}∐X_{2},Y_{1}∐Y_{2}}^{\left(o\right)}$ be the projection onto the $R_{X_{1}∐X_{2},Y_{1}∐Y_{2}}^{\left(o\right)}$-equivalence classes. Define the mapping $f:{\mathrm{DMC}}_{X_{1},Y_{1}}\times {\mathrm{DMC}}_{X_{2},Y_{2}}\to {\mathrm{DMC}}_{X_{1}∐X_{2},Y_{1}∐Y_{2}}^{\left(o\right)}$ as $f\left(W_{1},W_{2}\right)=\mathrm{Proj}\left(W_{1}⊕W_{2}\right)$. Clearly, *f* is continuous.

Now, define the equivalence relation *R* on ${\mathrm{DMC}}_{X_{1},Y_{1}}\times {\mathrm{DMC}}_{X_{2},Y_{2}}$ as:

$$\left(W_{1},W_{2}\right)R\left(W_{1}^{\prime },W_{2}^{\prime }\right)\;\;\Leftrightarrow \;\;W_{1}R_{X_{1},Y_{1}}^{\left(o\right)}W_{1}^{\prime }\;\mathrm{and}\;W_{2}R_{X_{2},Y_{2}}^{\left(o\right)}W_{2}^{\prime }.$$

The discussion before the proposition shows that $f\left(W_{1},W_{2}\right)=\mathrm{Proj}\left(W_{1}⊕W_{2}\right)$ depends only on the *R*-equivalence class of $\left(W_{1},W_{2}\right)$. Lemma 2 now shows that the transcendent map of *f* defined on $({\mathrm{DMC}}_{X_{1},Y_{1}}\times {\mathrm{DMC}}_{X_{2},Y_{2}})/R$ is continuous.

Notice that $({\mathrm{DMC}}_{X_{1},Y_{1}}\times {\mathrm{DMC}}_{X_{2},Y_{2}})/R$ can be identified with ${\mathrm{DMC}}_{X_{1},Y_{1}}^{\left(o\right)}\times {\mathrm{DMC}}_{X_{2},Y_{2}}^{\left(o\right)}$. Therefore, we can define *f* on ${\mathrm{DMC}}_{X_{1},Y_{1}}^{\left(o\right)}\times {\mathrm{DMC}}_{X_{2},Y_{2}}^{\left(o\right)}$ through this identification. Moreover, since ${\mathrm{DMC}}_{X_{1},Y_{1}}$ and ${\mathrm{DMC}}_{X_{2},Y_{2}}^{\left(o\right)}$ are locally compact and Hausdorff, Corollary 1 implies that the canonical bijection between $({\mathrm{DMC}}_{X_{1},Y_{1}}\times {\mathrm{DMC}}_{X_{2},Y_{2}})/R$ and ${\mathrm{DMC}}_{X_{1},Y_{1}}^{\left(o\right)}\times {\mathrm{DMC}}_{X_{2},Y_{2}}^{\left(o\right)}$ is a homeomorphism.

Now, since the mapping *f* on ${\mathrm{DMC}}_{X_{1},Y_{1}}^{\left(o\right)}\times {\mathrm{DMC}}_{X_{2},Y_{2}}^{\left(o\right)}$ is just the channel sum, we conclude that the mapping $\left({\hat{W}}_{1},{\bar{W}}_{2}\right)\to {\hat{W}}_{1}⊕{\bar{W}}_{2}$ from ${\mathrm{DMC}}_{X_{1},Y_{1}}^{\left(o\right)}\times {\mathrm{DMC}}_{X_{2},Y_{2}}^{\left(o\right)}$ to ${\mathrm{DMC}}_{X_{1}∐X_{2},Y_{1}∐Y_{2}}^{\left(o\right)}$ is continuous. ☐

## 6. Continuity in the Strong Topology

The following lemma provides a way to check whether a mapping defined on $\left({\mathrm{DMC}}_{X,*}^{\left(o\right)},T_{s,X,*}^{\left(o\right)}\right)$ is continuous:

**Lemma** **6.** *Let (S,V) be an arbitrary topological space. A mapping $f:{\mathrm{DMC}}_{X,*}^{\left(o\right)}\to S$ is continuous on $\left({\mathrm{DMC}}_{X,*}^{\left(o\right)},T_{s,X,*}^{\left(o\right)}\right)$ if and only if it is continuous on $\left({\mathrm{DMC}}_{X,[n]}^{\left(o\right)},T_{X,[n]}^{\left(o\right)}\right)$ for every n≥1.*

**Proof.**  $$\begin{array}{cc} f\;\mathrm{is}\;\mathrm{continuous}\;\mathrm{on}\;({\mathrm{DMC}}_{X,*}^{\left(o\right)},T_{s,X,*}^{\left(o\right)})\;\; & \Leftrightarrow \;\;f^{-1}\left(V\right)\in T_{s,X,*}^{\left(o\right)}\;\;\forall V\in V\\ & \Leftrightarrow \;\;f^{-1}\left(V\right)\cap {\mathrm{DMC}}_{X,[n]}^{\left(o\right)}\in T_{X,[n]}^{\left(o\right)}\;\;\forall n\geq 1,\;\forall V\in V\\ & \Leftrightarrow \;\;f\;\mathrm{is}\;\mathrm{continuous}\;\mathrm{on}\;({\mathrm{DMC}}_{X,[n]}^{\left(o\right)},T_{X,[n]}^{\left(o\right)})\;\;\forall n\geq 1. \end{array}$$

☐

Since the channel parameters *I*, *C*, $P_{e}$, *Z*, $P_{e,C}$ and $P_{e,n,M}$ are defined on ${\mathrm{DMC}}_{X,[n]}^{\left(o\right)}$ for every n≥1 (see Section 5), they are also defined on ${\mathrm{DMC}}_{X,*}^{\left(o\right)}=\underset{n\geq 1}{⋃}{\mathrm{DMC}}_{X,[n]}^{\left(o\right)}$. The following proposition shows that those parameters are continuous in the strong topology:

**Proposition** **6.** *Let $U_{X}$ be the standard topology on $\Delta_{X}$. We have:*

- *$I:\Delta_{X}\times {\mathrm{DMC}}_{X,*}^{\left(o\right)}\to R^{+}$ is continuous on $\left(\Delta_{X}\times {\mathrm{DMC}}_{X,*}^{\left(o\right)},U_{X}⊗T_{s,X,*}^{\left(o\right)}\right)$ and concave in p.*
- *$C:{\mathrm{DMC}}_{X,*}^{\left(o\right)}\to R^{+}$ is continuous on $\left({\mathrm{DMC}}_{X,*}^{\left(o\right)},T_{s,X,*}^{\left(o\right)}\right)$.*
- *$P_{e}:\Delta_{X}\times {\mathrm{DMC}}_{X,*}^{\left(o\right)}\to \left[0,1\right]$ is continuous on $\left(\Delta_{X}\times {\mathrm{DMC}}_{X,*}^{\left(o\right)},U_{X}⊗T_{s,X,*}^{\left(o\right)}\right)$ and concave in p.*
- *$Z:{\mathrm{DMC}}_{X,*}^{\left(o\right)}\to \left[0,1\right]$ is continuous on $\left({\mathrm{DMC}}_{X,*}^{\left(o\right)},T_{s,X,*}^{\left(o\right)}\right)$.*
- *For every code C on X, $P_{e,C}:{\mathrm{DMC}}_{X,*}^{\left(o\right)}\to \left[0,1\right]$ is continuous on $\left({\mathrm{DMC}}_{X,*}^{\left(o\right)},T_{s,X,*}^{\left(o\right)}\right)$.*
- *For every n>0 and every $1\leq M\leq {\left|X\right|}^{n}$, the mapping $P_{e,n,M}:{\mathrm{DMC}}_{X,*}^{\left(o\right)}\to \left[0,1\right]$ is continuous on $\left({\mathrm{DMC}}_{X,*}^{\left(o\right)},T_{s,X,*}^{\left(o\right)}\right)$.*

**Proof.**  The continuity of $C,Z,P_{e,C}$ and $P_{e,n,M}$ immediately follows from Proposition 4 and Lemma 6. Since the proofs of the continuity of *I* and *Z* are similar, we only prove the continuity for *I*.

Due to the distributivity of the product with respect to disjoint unions, we have:

$$\begin{array}{c} \Delta_{X}\times {\mathrm{DMC}}_{X,*}=\underset{n\geq 1}{∐}\left(\Delta_{X}\times {\mathrm{DMC}}_{X,[n]}\right), \end{array}$$

and:

$$\begin{array}{c} U_{X}⊗T_{s,X,*}=\underset{n\geq 1}{⨁}\left(U_{X}⊗T_{X,[n]}\right). \end{array}$$

Therefore, $\left(\Delta_{X}\times {\mathrm{DMC}}_{X,*},U_{X}⊗T_{s,X,*}\right)$ is the disjoint union of the spaces ${\left(\Delta_{X}\times {\mathrm{DMC}}_{X,[n]}\right)}_{n\geq 1}$. Moreover, *I* is continuous on $\Delta_{X}\times {\mathrm{DMC}}_{X,[n]}$ for every n≥1. We conclude that *I* is continuous on $\left(\Delta_{X}\times {\mathrm{DMC}}_{X,*},U_{X}⊗T_{s,X,*}\right)$.

Define the relation *R* on $\Delta_{X}\times {\mathrm{DMC}}_{X,*}$ as follows: $\left(p_{1},W_{1}\right)R\left(p_{2},W_{2}\right)$ if and only if $p_{1}=p_{2}$ and $W_{1}R_{X,*}^{\left(o\right)}W_{2}$. Since I(p,W) depends only on the *R*-equivalence class of (p,W), Lemma 2 shows that the transcendent map of *I* is a continuous mapping from $\left(\left(\Delta_{X}\times {\mathrm{DMC}}_{X,*}\right)/R,\left(U_{X}⊗T_{s,X,*}\right)/R\right)$ to $R^{+}$. On the other hand, since $\Delta_{X}$ is locally compact and Hausdorff, Theorem 1 implies that $\left(\left(\Delta_{X}\times {\mathrm{DMC}}_{X,*}\right)/R,\left(U_{X}⊗T_{s,X,*}\right)/R\right)$ can be identified with $\left(\Delta_{X}\times \left({\mathrm{DMC}}_{X,*}/R_{X,*}^{\left(o\right)}\right),U_{X}⊗\left(T_{s,X,*}/R_{X,*}^{\left(o\right)}\right)\right)=\left(\Delta_{X}\times {\mathrm{DMC}}_{X,*}^{\left(o\right)},U_{X}⊗T_{s,X,*}^{\left(o\right)}\right)$. Therefore, *I* is continuous on $\left(\Delta_{X}\times {\mathrm{DMC}}_{X,*}^{\left(o\right)},U_{X}⊗T_{s,X,*}^{\left(o\right)}\right)$. ☐

It is also possible to extend the definition of all the channel operations that were defined in Section 5 to ${\mathrm{DMC}}_{X,*}^{\left(o\right)}$. Moreover, it is possible to show that many channel operations are continuous in the strong topology:

**Proposition** **7.** *Assume that all equivalent channel spaces are endowed with the strong topology. We have:*

- *The mapping $\left({\hat{W}}_{1},{\bar{W}}_{2}\right)\to {\hat{W}}_{1}⊕{\bar{W}}_{2}$ from ${\mathrm{DMC}}_{X_{1},*}^{\left(o\right)}\times {\mathrm{DMC}}_{X_{2},Y_{2}}^{\left(o\right)}$ to ${\mathrm{DMC}}_{X_{1}∐X_{2},*}^{\left(o\right)}$ is continuous.*
- *The mapping $\left({\hat{W}}_{1},{\bar{W}}_{2}\right)\to {\hat{W}}_{1}⊗{\bar{W}}_{2}$ from ${\mathrm{DMC}}_{X_{1},*}^{\left(o\right)}\times {\mathrm{DMC}}_{X_{2},Y_{2}}^{\left(o\right)}$ to ${\mathrm{DMC}}_{X_{1}\times X_{2},*}^{\left(o\right)}$ is continuous.*
- *The mapping $\left({\hat{W}}_{1},{\bar{W}}_{2},\alpha \right)\to \left[\alpha {\hat{W}}_{1},\left(1-\alpha \right){\bar{W}}_{2}\right]$ from ${\mathrm{DMC}}_{X,*}\times {\mathrm{DMC}}_{X,Y_{2}}^{\left(o\right)}\times \left[0,1\right]$ to ${\mathrm{DMC}}_{X,*}^{\left(o\right)}$ is continuous.*
- *For any binary operation ∗ on X, the mapping $\hat{W}\to {\hat{W}}^{-}$ from ${\mathrm{DMC}}_{X,*}^{\left(o\right)}$ to ${\mathrm{DMC}}_{X,*}^{\left(o\right)}$ is continuous.*
- *For any binary operation ∗ on X, the mapping $\hat{W}\to {\hat{W}}^{+}$ from ${\mathrm{DMC}}_{X,*}^{\left(o\right)}$ to ${\mathrm{DMC}}_{X,*}^{\left(o\right)}$ is continuous.*

**Proof.**  We only prove the continuity of the channel interpolation because the proof of the continuity of other operations is similar.

Let U be the standard topology on [0,1]. Due to the distributivity of the product with respect to disjoint unions, we have:

$${\mathrm{DMC}}_{X,*}\times {\mathrm{DMC}}_{X,Y_{2}}\times \left[0,1\right]=\underset{n\geq 1}{∐}\left({\mathrm{DMC}}_{X,[n]}\times {\mathrm{DMC}}_{X,Y_{2}}\times \left[0,1\right]\right),$$

and:

$$T_{s,X,*}⊗T_{X,Y_{2}}⊗U=\underset{n\geq 1}{⨁}\left(T_{X,[n]}⊗T_{X,Y_{2}}⊗U\right).$$

Therefore, the space ${\mathrm{DMC}}_{X,*}\times {\mathrm{DMC}}_{X,Y_{2}}\times \left[0,1\right]$ is the topological disjoint union of the spaces ${\left({\mathrm{DMC}}_{X,[n]}\times {\mathrm{DMC}}_{X,Y_{2}}\times \left[0,1\right]\right)}_{n\geq 1}$.

For every n≥1, let ${\mathrm{Proj}}_{n}$ be the projection onto the $R_{X,\left[n\right]∐Y_{2}}^{\left(o\right)}$-equivalence classes, and let $i_{n}$ be the canonical injection from ${\mathrm{DMC}}_{X,\left[n\right]∐Y_{2}}^{\left(o\right)}$ to ${\mathrm{DMC}}_{X,*}^{\left(o\right)}$.

Define the mapping $f:{\mathrm{DMC}}_{X,*}\times {\mathrm{DMC}}_{X,Y_{2}}\times \left[0,1\right]\to {\mathrm{DMC}}_{X,*}^{\left(o\right)}$ as:

$$f\left(W_{1},W_{2},\alpha \right)=i_{n}\left({\mathrm{Proj}}_{n}\left(\left[\alpha W_{1},\left(1-\alpha \right)W_{2}\right]\right)\right)=\left[\alpha {\hat{W}}_{1},\left(1-\alpha \right){\bar{W}}_{2}\right],$$

where *n* is the unique integer satisfying $W_{1}\in {\mathrm{DMC}}_{X,[n]}$. ${\hat{W}}_{1}$ and ${\bar{W}}_{2}$ are the $R_{X,[n]}^{\left(o\right)}$ and $R_{X,Y_{2}}^{\left(o\right)}$-equivalence classes of $W_{1}$ and $W_{2}$, respectively.

Due to Proposition 3 and due to the continuity of ${\mathrm{Proj}}_{n}$ and $i_{n}$, the mapping *f* is continuous on ${\mathrm{DMC}}_{X,[n]}\times {\mathrm{DMC}}_{X,Y_{2}}\times \left[0,1\right]$ for every n≥1. Therefore, *f* is continuous on $\left({\mathrm{DMC}}_{X,*}\times {\mathrm{DMC}}_{X,Y_{2}}\times \left[0,1\right],T_{s,X,*}⊗T_{X,Y_{2}}⊗U\right)$.

Let $R^{\prime }$ be the equivalence relation defined on ${\mathrm{DMC}}_{X,*}\times {\mathrm{DMC}}_{X,Y_{2}}$ as follows: $\left(W_{1},W_{2}\right)R^{\prime }\left(W_{1}^{\prime },W_{2}^{\prime }\right)$ if and only if $W_{1}R_{X,*}^{\left(o\right)}W_{1}^{\prime }$ and $W_{2}R_{X,Y_{2}}^{\left(o\right)}W_{2}^{\prime }$. Furthermore, define the equivalence relation *R* on ${\mathrm{DMC}}_{X,*}\times {\mathrm{DMC}}_{X,Y_{2}}\times \left[0,1\right]$ as follows: $\left(W_{1},W_{2},\alpha \right)R\left(W_{1}^{\prime },W_{2}^{\prime },\alpha^{\prime }\right)$ if and only if $\left(W_{1},W_{2}\right)R^{\prime }\left(W_{1}^{\prime },W_{2}^{\prime }\right)$ and $\alpha =\alpha^{\prime }$.

Since $f(W_{1},W_{2},\alpha )$ depends only on the *R*-equivalence class of $\left(W_{1},W_{2},\alpha \right)$, Lemma 2 implies that the transcendent mapping of *f* is continuous on $({\mathrm{DMC}}_{X,*}\times {\mathrm{DMC}}_{X,Y_{2}}\times \left[0,1\right])/R$.

Since [0,1] is Hausdorff and locally compact, Theorem 1 implies that the canonical bijection from $({\mathrm{DMC}}_{X,*}\times {\mathrm{DMC}}_{X,Y_{2}}\times \left[0,1\right])/R$ to $\left(\left({\mathrm{DMC}}_{X,*}\times {\mathrm{DMC}}_{X,Y_{2}}\right)/R^{\prime }\right)\times \left[0,1\right])$ is a homeomorphism. On the other hand, since $\left({\mathrm{DMC}}_{X,*},T_{s,X,*}\right)$ and ${\mathrm{DMC}}_{X,Y_{2}}^{\left(o\right)}={\mathrm{DMC}}_{X,Y_{2}}/R_{X,Y_{2}}^{\left(o\right)}$ are Hausdorff and locally compact, Corollary 1 implies that the canonical bijection from ${\mathrm{DMC}}_{X,*}^{\left(o\right)}\times {\mathrm{DMC}}_{X,Y_{2}}^{\left(o\right)}$ to $\left({\mathrm{DMC}}_{X,*}\times {\mathrm{DMC}}_{X,Y_{2}}\right)/R^{\prime }$ is a homeomorphism. We conclude that the channel interpolation is continuous on $\left({\mathrm{DMC}}_{X,*}^{\left(o\right)}\times {\mathrm{DMC}}_{X,Y_{2}}^{\left(o\right)}\times \left[0,1\right],T_{s,X,*}^{\left(o\right)}⊗T_{X,Y}^{\left(o\right)}⊗U\right)$. ☐

**Corollary** **3.** *$\left({\mathrm{DMC}}_{X,*}^{\left(o\right)},T_{s,X,*}^{\left(o\right)}\right)$ is strongly contractible to every point in ${\mathrm{DMC}}_{X,*}^{\left(o\right)}$.*

**Proof.**  Fix ${\hat{W}}_{0}\in {\mathrm{DMC}}_{X,*}^{\left(o\right)}$. Define the mapping $H:{\mathrm{DMC}}_{X,*}^{\left(o\right)}\times \left[0,1\right]\to {\mathrm{DMC}}_{X,*}^{\left(o\right)}$ as $H\left(\hat{W},\alpha \right)=\left[\alpha {\hat{W}}_{0},\left(1-\alpha \right)\hat{W}\right]$. *H* is continuous by Proposition 7. We also have $H\left(\hat{W},0\right)=\hat{W}$ and $H\left(\hat{W},1\right)={\hat{W}}_{0}$ for every $\hat{W}\in {\mathrm{DMC}}_{X,*}^{\left(o\right)}$. Moreover, $H\left({\hat{W}}_{0},\alpha \right)={\hat{W}}_{0}$ for every 0≤α≤1. Therefore, $\left({\mathrm{DMC}}_{X,*}^{\left(o\right)},T_{s,X,*}^{\left(o\right)}\right)$ is strongly contractible to every point in ${\mathrm{DMC}}_{X,*}^{\left(o\right)}$. ☐

The reader might be wondering why channel operations such as the channel sum were not shown to be continuous on the whole space ${\mathrm{DMC}}_{X_{1},*}^{\left(o\right)}\times {\mathrm{DMC}}_{X_{2},*}^{\left(o\right)}$ instead of the smaller space ${\mathrm{DMC}}_{X_{1},*}^{\left(o\right)}\times {\mathrm{DMC}}_{X_{2},Y_{2}}^{\left(o\right)}$. The reason is because we cannot apply Corollary 1 to ${\mathrm{DMC}}_{X_{1},*}\times {\mathrm{DMC}}_{X_{2},*}$ and ${\mathrm{DMC}}_{X_{1},*}^{\left(o\right)}\times {\mathrm{DMC}}_{X_{2},*}^{\left(o\right)}$ since neither ${\mathrm{DMC}}_{X_{1},*}^{\left(o\right)}$, nor ${\mathrm{DMC}}_{X_{2},*}^{\left(o\right)}$ is locally compact (under the strong topology).

One potential method to show the continuity of the channel sum on $\left({\mathrm{DMC}}_{X_{1},*}^{\left(o\right)}\times {\mathrm{DMC}}_{X_{2},*}^{\left(o\right)},T_{s,X_{1},*}^{\left(o\right)}⊗T_{s,X_{2},*}^{\left(o\right)}\right)$ is as follows: let *R* be the equivalence relation on ${\mathrm{DMC}}_{X_{1},*}\times {\mathrm{DMC}}_{X_{2},*}$ defined as $\left(W_{1},W_{2}\right)R\left(W_{1}^{\prime },W_{2}^{\prime }\right)$ if and only if $W_{1}R_{X_{1},*}^{\left(o\right)}W_{1}^{\prime }$ and $W_{2}R_{X_{2},*}^{\left(o\right)}W_{2}^{\prime }$. We can identify $({\mathrm{DMC}}_{X_{1},*}\times {\mathrm{DMC}}_{X_{2},*})/R$ with ${\mathrm{DMC}}_{X_{1},*}^{\left(o\right)}\times {\mathrm{DMC}}_{X_{2},*}^{\left(o\right)}$ through the canonical bijection. Using Lemma 2, it is easy to see that the mapping $\left({\hat{W}}_{1},{\bar{W}}_{2}\right)\to {\hat{W}}_{1}⊕{\bar{W}}_{2}$ is continuous from $\left({\mathrm{DMC}}_{X_{1},*}^{\left(o\right)}\times {\mathrm{DMC}}_{X_{2},*}^{\left(o\right)},\left(T_{s,X_{1},*}⊗T_{s,X_{2},*}\right)/R\right)$ to $\left({\mathrm{DMC}}_{X_{1}∐X_{2},*}^{\left(o\right)},T_{s,X_{1}∐X_{2},*}^{\left(o\right)}\right)$.

It was shown in [17] that the topology $(T_{s,X_{1},*}⊗T_{s,X_{2},*})/R$ is homeomorphic to $\kappa (T_{s,X_{1},*}^{\left(o\right)}⊗T_{s,X_{2},*}^{\left(o\right)})$ through the canonical bijection, where $\kappa (T_{s,X_{1},*}^{\left(o\right)}⊗T_{s,X_{2},*}^{\left(o\right)})$ is the coarsest topology that is both compactly generated and finer than $T_{s,X_{1},*}^{\left(o\right)}⊗T_{s,X_{2},*}^{\left(o\right)}$. Therefore, the mapping $\left({\hat{W}}_{1},{\bar{W}}_{2}\right)\to {\hat{W}}_{1}⊕{\bar{W}}_{2}$ is continuous on $\left({\mathrm{DMC}}_{X_{1},*}^{\left(o\right)}\times {\mathrm{DMC}}_{X_{2},*}^{\left(o\right)},\kappa \left(T_{s,X_{1},*}^{\left(o\right)}⊗T_{s,X_{2},*}^{\left(o\right)}\right)\right)$. This means that if $T_{s,X_{1},*}^{\left(o\right)}⊗T_{s,X_{2},*}^{\left(o\right)}$ is compactly generated, we will have $T_{s,X_{1},*}^{\left(o\right)}⊗T_{s,X_{2},*}^{\left(o\right)}=\kappa \left(T_{s,X_{1},*}^{\left(o\right)}⊗T_{s,X_{2},*}^{\left(o\right)}\right)$, and so, the channel sum will be continuous on $\left({\mathrm{DMC}}_{X_{1},*}^{\left(o\right)}\times {\mathrm{DMC}}_{X_{2},*}^{\left(o\right)},T_{s,X_{1},*}^{\left(o\right)}⊗T_{s,X_{2},*}^{\left(o\right)}\right)$. Note that although $T_{s,X_{1},*}^{\left(o\right)}$ and $T_{s,X_{2},*}^{\left(o\right)}$ are compactly generated, their product $T_{s,X_{1},*}^{\left(o\right)}⊗T_{s,X_{2},*}^{\left(o\right)}$ might not be compactly generated.

## 7. Continuity in the Noisiness/Weak-∗ and the Total Variation Topologies

We need to express the channel parameters and operations in terms of the Blackwell measures.

### 7.1. Channel Parameters

The following proposition shows that many channel parameters can be expressed as an integral of a continuous function with respect to the Blackwell measure:

**Proposition** **8.** *For every $\hat{W}\in {\mathrm{DMC}}_{X,*}^{\left(o\right)}$, we have:*

$$\forall p\in \Delta_{X},\;I\left(p,\hat{W}\right)=H\left(p\right)-\left|X\right|\cdot \int_{\Delta_{X}}\left(\sum_{x\in X}p\left(x\right)p^{\prime }\left(x\right)\log\frac{p\left(x\right)p^{\prime }\left(x\right)}{\sum_{x^{\prime }}p\left(x^{\prime }\right)p^{\prime }\left(x^{\prime }\right)}\right)\cdot d{\mathrm{MP}}_{\hat{W}}\left(p^{\prime }\right),$$

$$\forall p\in \Delta_{X},\;P_{e}\left(p,\hat{W}\right)=1-\left|X\right|\int_{\Delta_{X}}\max_{x\in X}\left\{p\left(x\right)\times p^{\prime }\left(x\right)\right\}\cdot d{\mathrm{MP}}_{\hat{W}}\left(p^{\prime }\right),$$

$$\mathrm{if}\;|X|\geq 2,\;Z\left(\hat{W}\right)=\frac{1}{|X|-1}\sum_{\begin{array}{c} x,x^{\prime }\in X,\\ x\neq x^{\prime } \end{array}}\int_{\Delta_{X}}\sqrt{p\left(x\right)p\left(x^{\prime }\right)}\cdot d{\mathrm{MP}}_{\hat{W}}\left(p\right),$$

$$\mathrm{For}\;\mathrm{every}\;\mathrm{code}\;C\subset X^{n},\;P_{e,C}\left(\hat{W}\right)=1-\frac{{\left|X\right|}^{n}}{\left|C\right|}\int_{\Delta_{X}^{n}}\max_{x_{1}^{n}\in C}\left\{\prod_{i=1}^{n}p_{i}\left(x_{i}\right)\right\}d{\mathrm{MP}}_{\hat{W}}^{n}\left(p_{1}^{n}\right),$$

*where H(p) is the entropy of p and ${\mathrm{MP}}_{\hat{W}}^{n}$ is the product measure on $\Delta_{X}^{n}$ obtained by multiplying ${\mathrm{MP}}_{\hat{W}}$ with itself n times. Note that we adopt the standard convention that $0\log\frac{0}{0}=0$.*

**Proof.**  By choosing any representative channel $W\in \hat{W}$ and replacing W(y|x) by $\left|X\right|P_{W}^{o}\left(y\right)W_{y}^{-1}\left(x\right)$ in the definitions of the channel parameters, all the above formulas immediately follow. Let us show how this works for $P_{e}$:

Pe(p,W^)=Pe(p,W)=(a)1−∑y∈Im(W)maxx∈X{p(x)W(y|x)}=1−∑y∈Im(W)maxx∈Xp(x)·|X|·PWo(y)Wy−1(x)=1−|X|∑y∈Im(W)maxx∈X{p(x)Wy−1(x)}·PWo(y)=1−|X|∫ΔXmaxx∈X{p(x)p′(x)}·dMPW(p′)=1−|X|∫ΔXmaxx∈X{p(x)p′(x)}·dMPW^(p′),

where (a) is true because W(y|x)=0 for y∉Im(W). ☐

**Proposition** **9.** *Let $U_{X}$ be the standard topology on $\Delta_{X}$. We have:*

- *$I:\Delta_{X}\times {\mathrm{DMC}}_{X,*}^{\left(o\right)}\to R^{+}$ is continuous on $\left(\Delta_{X}\times {\mathrm{DMC}}_{X,*}^{\left(o\right)},U_{X}⊗T_{X,*}^{\left(o\right)}\right)$ and concave in p.*
- *$C:{\mathrm{DMC}}_{X,*}^{\left(o\right)}\to R^{+}$ is continuous on $\left({\mathrm{DMC}}_{X,*}^{\left(o\right)},T_{X,*}^{\left(o\right)}\right)$.*
- *$P_{e}:\Delta_{X}\times {\mathrm{DMC}}_{X,*}^{\left(o\right)}\to \left[0,1\right]$ is continuous on $\left(\Delta_{X}\times {\mathrm{DMC}}_{X,*}^{\left(o\right)},U_{X}⊗T_{X,*}^{\left(o\right)}\right)$ and concave in p.*
- *$Z:{\mathrm{DMC}}_{X,*}^{\left(o\right)}\to \left[0,1\right]$ is continuous on $\left({\mathrm{DMC}}_{X,*}^{\left(o\right)},T_{X,*}^{\left(o\right)}\right)$.*
- *For every code C on X, $P_{e,C}:{\mathrm{DMC}}_{X,*}^{\left(o\right)}\to \left[0,1\right]$ is continuous on $\left({\mathrm{DMC}}_{X,*}^{\left(o\right)},T_{X,*}^{\left(o\right)}\right)$.*
- *For every n>0 and every $1\leq M\leq {\left|X\right|}^{n}$, the mapping $P_{e,n,M}:{\mathrm{DMC}}_{X,*}^{\left(o\right)}\to \left[0,1\right]$ is continuous on $\left({\mathrm{DMC}}_{X,*}^{\left(o\right)},T_{X,*}^{\left(o\right)}\right)$.*

**Proof.**  We associate the space MP(X) with the weak-∗ topology. Define the mapping:

$$\bar{I}:\Delta_{X}\times \mathrm{MP}\left(X\right)\to R^{+}$$

as follows:

$$\bar{I}\left(p,\mathrm{MP}\right)=H\left(p\right)-\left|X\right|\cdot \int_{\Delta_{X}}\left(\sum_{x\in X}p\left(x\right)p^{\prime }\left(x\right)\log\frac{p\left(x\right)p^{\prime }\left(x\right)}{\sum_{x^{\prime }}p\left(x^{\prime }\right)p^{\prime }\left(x^{\prime }\right)}\right)\cdot d\mathrm{MP}\left(p^{\prime }\right),$$

Lemma 5 implies that $\bar{I}$ is continuous. On the other hand, Proposition 8 shows that $I\left(p,\hat{W}\right)=\bar{I}\left(p,{\mathrm{MP}}_{\hat{W}}\right)$. Therefore, *I* is continuous on $\left(\Delta_{X}\times {\mathrm{DMC}}_{X,*}^{\left(o\right)},U_{X}⊗T_{X,*}^{\left(o\right)}\right)$. We can prove the continuity of $P_{e}$ and *Z* similarly.

Now, define the mapping $\bar{C}:\mathrm{MP}\left(X\right)\to R$ as:

$$\bar{C}\left(\mathrm{MP}\right)=\underset{p\in \Delta_{X}}{\sup}\bar{I}\left(p,\mathrm{MP}\right).$$

Fix MP∈MP(X), and let ϵ>0. Since MP(X) is compact (under the weak-∗ topology), Lemma 1 implies the existence of a weakly-∗ open neighborhood $U_{\mathrm{MP}}$ of MP such that $|\bar{I}\left(p,\mathrm{MP}\right)-\bar{I}\left(p,{\mathrm{MP}}^{\prime }\right)|<\epsilon$ for every ${\mathrm{MP}}^{\prime }\in U_{\mathrm{MP}}$ and every $p\in \Delta_{X}$. Therefore, for every ${\mathrm{MP}}^{\prime }\in U_{\mathrm{MP}}$ and every $p\in \Delta_{X}$, we have:

$$\bar{I}\left(p,\mathrm{MP}\right)<\bar{I}\left(p,{\mathrm{MP}}^{\prime }\right)+\epsilon \leq \bar{C}\left({\mathrm{MP}}^{\prime }\right)+\epsilon ,$$

hence,

$$\bar{C}\left(\mathrm{MP}\right)=\underset{p\in \Delta_{X}}{\sup}\bar{I}\left(p,\mathrm{MP}\right)\leq \bar{C}\left({\mathrm{MP}}^{\prime }\right)+\epsilon .$$

Similarly, we can show that $\bar{C}\left({\mathrm{MP}}^{\prime }\right)\leq \bar{C}\left(\mathrm{MP}\right)+\epsilon$. This shows that $|\bar{C}\left({\mathrm{MP}}^{\prime }\right)-\bar{C}\left(\mathrm{MP}\right)|\leq \;\epsilon$ for every ${\mathrm{MP}}^{\prime }\in U_{\mathrm{MP}}$. Therefore, $\bar{C}$ is continuous. However, $C\left(\hat{W}\right)=\bar{C}\left({\mathrm{MP}}_{\hat{W}}\right)$, so *C* is continuous on $\left({\mathrm{DMC}}_{X,*}^{\left(o\right)},T_{X,*}^{\left(o\right)}\right)$.

Now for every 0≤i≤n, define the mapping $f_{i}:\Delta_{X}^{i}\times \mathrm{MP}\left(X\right)\to R$ backward-recursively as follows:

- $f_{n}\left(p_{1}^{n},\mathrm{MP}\right)=\max_{x_{1}^{n}\in C}\left\{\prod_{i=1}^{n}p_{i}\left(x_{i}\right)\right\}$.
- For every 0≤i<n, define:
  $$f_{i}\left(p_{1}^{i},\mathrm{MP}\right)=\int_{\Delta_{X}}f_{i+1}\left(p_{1}^{i+1},\mathrm{MP}\right)\cdot d\mathrm{MP}\left(p_{i+1}\right).$$

Clearly $f_{n}$ is continuous. Now, let 0≤i<n, and assume that $f_{i+1}$ is continuous. If we let $S=\Delta_{X}^{i}\times \mathrm{MP}\left(X\right)$, Lemma 5 implies that the mapping $F_{i}:\Delta_{X}^{i}\times \mathrm{MP}\left(X\right)\times \mathrm{MP}\left(X\right)$ defined as:

$$F_{i}\left(p_{1}^{i},\mathrm{MP},{\mathrm{MP}}^{\prime }\right)=\int_{\Delta_{X}}f_{i+1}\left(p_{1}^{i+1},\mathrm{MP}\right)\cdot d{\mathrm{MP}}^{\prime }\left(p_{i+1}\right)$$

is continuous. However, $f_{i}\left(p_{1}^{i},\mathrm{MP}\right)=F_{i}\left(p_{1}^{i},\mathrm{MP},\mathrm{MP}\right)$, so $f_{i}$ is also continuous. Therefore, $f_{0}$ is continuous. By noticing that $P_{e,C}\left(\hat{W}\right)=1-\frac{{\left|X\right|}^{n}}{\left|C\right|}f_{0}\left({\mathrm{MP}}_{\hat{W}}\right)$, we conclude that $P_{e,C}$ is continuous on $\left({\mathrm{DMC}}_{X,*}^{\left(o\right)},T_{X,*}^{\left(o\right)}\right)$. Moreover, since $P_{e,n,M}$ is the minimum of a finite family of continuous mappings, it is continuous. ☐

It is worth mentioning that Proposition 6 can be shown from Proposition 9 because the noisiness topology is coarser than the strong topology.

**Corollary** **4.** *All the mappings in Proposition 9 are also continuous if we replace the noisiness topology $T_{X,*}^{\left(o\right)}$ with the total variation topology $T_{TV,X,*}^{\left(o\right)}$.*

**Proof.**  This is true because $T_{TV,X,*}^{\left(o\right)}$ is finer than $T_{X,*}^{\left(o\right)}$. ☐

### 7.2. Channel Operations

In the following, we show that we can express the channel operations in terms of Blackwell measures. We have all the tools to achieve this for the channel sum, channel product and channel interpolation. In order to express the channel polarization transformations in terms of the Blackwell measures, we need to introduce new definitions.

Let X be a finite set, and let ∗ be a binary operation on a finite set X. We say that ∗ is uniformity preserving if the mapping (a,b)→(a∗b,b) is a bijection from $X^{2}$ to itself [18]. For every a,b∈X, we denote the unique element c∈X satisfying c∗b=a as $c=a{/}^{*}b$. Note that ${/}^{*}$ is a binary operation, and it is uniformity preserving. ${/}^{*}$ is called the right-inverse of ∗. It was shown in [11] that a binary operation is polarizing if and only if it is uniformity preserving and its inverse is strongly ergodic.

Binary operations that are not uniformity preserving are not interesting for polarization theory because they do not preserve the symmetric capacity [11]. Therefore, we will only focus on polarization transformations that are based on uniformity preserving operations.

Let ∗ be a fixed uniformity preserving operation on X. Define the mapping $C^{-,*}:\Delta_{X}\times \Delta_{X}\to \Delta_{X}$ as

$$\left(C^{-,*}\left(p_{1},p_{2}\right)\right)\left(u_{1}\right)=\sum_{u_{2}\in X}p_{1}\left(u_{1}*u_{2}\right)p_{2}\left(u_{2}\right).$$

The probability distribution $C^{-,*}\left(p_{1},p_{2}\right)$ can be interpreted as follows: let $X_{1}$ and $X_{2}$ be two independent random variables in X that are distributed as $p_{1}$ and $p_{2}$, respectively, and let $\left(U_{1},U_{2}\right)$ be the random pair in $X^{2}$ defined as $\left(U_{1},U_{2}\right)=\left(X_{1}{/}^{*}X_{2},X_{2}\right)$, or equivalently $\left(X_{1},X_{2}\right)=\left(U_{1}*U_{2},U_{2}\right)$. $C^{-,*}\left(p_{1},p_{2}\right)$ is the probability distribution of $U_{1}$.

Clearly, $C^{-,*}$ is continuous. Therefore, the push-forward mapping $C_{\#}^{-,*}$ is continuous from $P(\Delta_{X}\times \Delta_{X})$ to $P\left(\Delta_{X}\right)=\mathrm{MP}\left(X\right)$ under both the weak-∗ and the total variation topologies (see Section 2.6). For every ${\mathrm{MP}}_{1},{\mathrm{MP}}_{2}\in \mathrm{MP}\left(X\right)$, we define the (−,∗)-convolution of ${\mathrm{MP}}_{1}$ and ${\mathrm{MP}}_{2}$ as:

$${\left({\mathrm{MP}}_{1},{\mathrm{MP}}_{2}\right)}^{-,*}=C_{\#}^{-,*}\left({\mathrm{MP}}_{1}\times {\mathrm{MP}}_{2}\right)\in \mathrm{MP}\left(X\right).$$

Since the product of meta-probability measures is continuous under both the weak-∗ and the total variation topologies (Appendix B and Appendix F), the (−,∗)-convolution is also continuous under these topologies.

For every $p_{1},p_{2}\in \Delta_{X}$ and every $u_{1}\in \mathrm{supp}\left(C^{-,*}\left(p_{1},p_{2}\right)\right)$, define $C^{+,u_{1},*}\left(p_{1},p_{2}\right)\in \Delta_{X}$ as:

$$\left(C^{+,u_{1},*}\left(p_{1},p_{2}\right)\right)\left(u_{2}\right)=\frac{p_{1}\left(u_{1}*u_{2}\right)p_{2}\left(u_{2}\right)}{\left(C^{-,*}\left(p_{1},p_{2}\right)\right)\left(u_{1}\right)}.$$

The probability distribution $C^{+,u_{1},*}\left(p_{1},p_{2}\right)$ can be interpreted as follows: if $X_{1},X_{2},U_{1}$ and $U_{2}$ are as above, $C^{+,u_{1},*}\left(p_{1},p_{2}\right)$ is the conditional probability distribution of $U_{2}$ given $U_{1}=u_{1}$.

Define the mapping $C^{+,*}:\Delta_{X}\times \Delta_{X}\to P\left(\Delta_{X}\right)=\mathrm{MP}\left(X\right)$ as follows:

$$C^{+,*}\left(p_{1},p_{2}\right)=\sum_{u_{1}\in \mathrm{supp}\left(C^{-,*}\left(p_{1},p_{2}\right)\right)}\left(C^{-,*}\left(p_{1},p_{2}\right)\right)\left(u_{1}\right)\cdot \delta_{C^{+,u_{1},*}\left(p_{1},p_{2}\right)},$$

where $\delta_{C^{+,u_{1},*}\left(p_{1},p_{2}\right)}$ is a Dirac measure centered at $C^{+,u_{1},*}\left(p_{1},p_{2}\right)$.

If $X_{1},X_{2},U_{1}$ and $U_{2}$ are as above, $C^{+,*}\left(p_{1},p_{2}\right)$ is the meta-probability measure that describes the possible conditional probability distributions of $U_{2}$ that are seen by someone having knowledge of $U_{1}$. Clearly, $C^{+,*}$ is a random mapping from $\Delta_{X}\times \Delta_{X}$ to $\Delta_{X}$. In Appendix H, we show that $C^{+,*}$ is a measurable random mapping. We also show in Appendix H that $C^{+,*}$ is a continuous mapping from $\Delta_{X}\times \Delta_{X}$ to MP(X) when the latter space is endowed with the weak-∗ topology. Lemmas 3 and 4 now imply that the push-forward mapping $C_{\#}^{+,*}$ is continuous under both the weak-∗ and the total variation topologies.

For every ${\mathrm{MP}}_{1},{\mathrm{MP}}_{2}\in \mathrm{MP}\left(X\right)$, we define the (+,∗)-convolution of ${\mathrm{MP}}_{1}$ and ${\mathrm{MP}}_{2}$ as:

$${\left({\mathrm{MP}}_{1},{\mathrm{MP}}_{2}\right)}^{+,*}=C_{\#}^{+,*}\left({\mathrm{MP}}_{1}\times {\mathrm{MP}}_{2}\right)\in \mathrm{MP}\left(X\right).$$

Since the product of meta-probability measures is continuous under both the weak-∗ and the total variation topologies (Appendix B and Appendix F), the (+,∗)-convolution is also continuous under these topologies.

**Proposition** **10.** *We have:*

- *For every ${\hat{W}}_{1}\in {\mathrm{DMC}}_{X_{1},*}^{\left(o\right)}$ and ${\bar{W}}_{2}\in {\mathrm{DMC}}_{X_{2},*}^{\left(o\right)}$, we have:*
  $${\mathrm{MP}}_{{\hat{W}}_{1}⊕{\bar{W}}_{2}}=\frac{|X_{1}|}{|X_{1}\left|+\right|X_{2}|}{\mathrm{MP}}_{{\hat{W}}_{1}}^{\prime }+\frac{|X_{2}|}{|X_{1}\left|+\right|X_{2}|}{\mathrm{MP}}_{{\bar{W}}_{2}}^{\prime },$$
  *where ${\mathrm{MP}}_{{\hat{W}}_{1}}^{\prime }$ (respectively ${\mathrm{MP}}_{{\hat{W}}_{2}}^{\prime }$) is the meta-push-forward of ${\mathrm{MP}}_{{\hat{W}}_{1}}$ (respectively ${\mathrm{MP}}_{{\hat{W}}_{2}}$) by the canonical injection from $X_{1}$ (respectively $X_{2}$) to $X_{1}∐X_{2}$.*
- *For every ${\hat{W}}_{1}\in {\mathrm{DMC}}_{X_{1},*}^{\left(o\right)}$ and ${\bar{W}}_{2}\in {\mathrm{DMC}}_{X_{2},*}^{\left(o\right)}$, we have:*
  $${\mathrm{MP}}_{{\hat{W}}_{1}⊗{\bar{W}}_{2}}={\mathrm{MP}}_{{\hat{W}}_{1}}⊗{\mathrm{MP}}_{{\bar{W}}_{2}}.$$
- *For every α∈[0,1] and every ${\hat{W}}_{1},{\hat{W}}_{2}\in {\mathrm{DMC}}_{X,*}^{\left(o\right)}$, we have:*
  $${\mathrm{MP}}_{\left[\alpha {\hat{W}}_{1},\left(1-\alpha \right){\hat{W}}_{2}\right]}=\alpha {\mathrm{MP}}_{{\hat{W}}_{1}}+\left(1-\alpha \right){\mathrm{MP}}_{{\hat{W}}_{2}}.$$
- *For every uniformity preserving binary operation ∗ on X, and every $\hat{W}\in {\mathrm{DMC}}_{X,*}^{\left(o\right)}$, we have:*
  $${\mathrm{MP}}_{{\hat{W}}^{-}}={\left({\mathrm{MP}}_{\hat{W}},{\mathrm{MP}}_{\hat{W}}\right)}^{-,*}.$$
- *For every uniformity preserving binary operation ∗ on X and every $\hat{W}\in {\mathrm{DMC}}_{X,*}^{\left(o\right)}$, we have:*
  $${\mathrm{MP}}_{{\hat{W}}^{+}}={\left({\mathrm{MP}}_{\hat{W}},{\mathrm{MP}}_{\hat{W}}\right)}^{+,*}.$$

**Proof.**  See Appendix I. ☐

Note that the polarization transformation formulas in Proposition 10 generalize the formulas given by Raginsky in [19] for binary-input channels.

**Proposition** **11.** *Assume that all equivalent channel spaces are endowed with the noisiness/weak-∗ or the total variation topology. We have:*

- *The mapping $\left({\hat{W}}_{1},{\bar{W}}_{2}\right)\to {\hat{W}}_{1}⊕{\bar{W}}_{2}$ from ${\mathrm{DMC}}_{X_{1},*}^{\left(o\right)}\times {\mathrm{DMC}}_{X_{2},*}^{\left(o\right)}$ to ${\mathrm{DMC}}_{X_{1}∐X_{2},*}^{\left(o\right)}$ is continuous.*
- *The mapping $\left({\hat{W}}_{1},{\bar{W}}_{2}\right)\to {\hat{W}}_{1}⊗{\bar{W}}_{2}$ from ${\mathrm{DMC}}_{X_{1},*}^{\left(o\right)}\times {\mathrm{DMC}}_{X_{2},*}^{\left(o\right)}$ to ${\mathrm{DMC}}_{X_{1}\times X_{2},*}^{\left(o\right)}$ is continuous.*
- *The mapping $\left({\hat{W}}_{1},{\bar{W}}_{2},\alpha \right)\to \left[\alpha {\hat{W}}_{1},\left(1-\alpha \right){\bar{W}}_{2}\right]$ from ${\mathrm{DMC}}_{X,*}\times {\mathrm{DMC}}_{X,*}^{\left(o\right)}\times \left[0,1\right]$ to ${\mathrm{DMC}}_{X,*}^{\left(o\right)}$ is continuous.*
- *For every uniformity preserving binary operation ∗ on X, the mapping $\hat{W}\to {\hat{W}}^{-}$ from ${\mathrm{DMC}}_{X,*}^{\left(o\right)}$ to ${\mathrm{DMC}}_{X,*}^{\left(o\right)}$ is continuous.*
- *For every uniformity preserving binary operation ∗ on X, the mapping $\hat{W}\to {\hat{W}}^{+}$ from ${\mathrm{DMC}}_{X,*}^{\left(o\right)}$ to ${\mathrm{DMC}}_{X,*}^{\left(o\right)}$ is continuous.*

**Proof.**  The proposition directly follows from Proposition 10 and the fact that all the meta-probability measure operations that are involved in the formulas are continuous under both the weak-∗ and the total variation topologies. ☐

**Corollary** **5.** *Both $\left({\mathrm{DMC}}_{X,*}^{\left(o\right)},T_{X,*}^{\left(o\right)}\right)$ and $\left({\mathrm{DMC}}_{X,*}^{\left(o\right)},T_{TV,X,*}^{\left(o\right)}\right)$ are strongly contractible to every point in ${\mathrm{DMC}}_{X,*}^{\left(o\right)}$.*

**Proof.**  We can use the same proof of Corollary 3. ☐

## 8. Discussion and Conclusions

Section 5 and Section 6 show that the quotient topology is relatively easy to work with. If one is interested in the space of equivalent channels sharing the same input and output alphabets, then using the quotient formulation of the topology seems to be the easiest way to prove theorems.

The continuity of the channel sum and the channel product on the whole product space $\left({\mathrm{DMC}}_{X_{1},*}^{\left(o\right)}\times {\mathrm{DMC}}_{X_{2},*}^{\left(o\right)},T_{s,X_{1},*}^{\left(o\right)}⊗T_{s,X_{2},*}^{\left(o\right)}\right)$ remains an open problem. As we mentioned in Section 6, it is sufficient to prove that the product topology $T_{s,X_{1},*}^{\left(o\right)}⊗T_{s,X_{2},*}^{\left(o\right)}$ is compactly generated.

## Abbreviations

The following abbreviations are used in this manuscript:

DMC	Discrete memoryless channel
TV	Total variation

## Appendix A. Proof of Lemma 1

Fix ϵ>0, and let (s,t)∈S×T. Since *f* is continuous, there exists a neighborhood $O_{s,t}$ of (s,t) in S×T such that for every $\left(s^{\prime },t^{\prime }\right)\in O_{s,t}$, we have $|f\left(s^{\prime },t^{\prime }\right)-f\left(s,t\right)|<\frac{\epsilon }{2}$. Moreover, since products of open sets form a base for the product topology, there exists an open neighborhood $V_{s,t}$ of *s* in (S,V) and an open neighborhood $U_{s,t}$ of *t* in *T* such that $V_{s,t}\times U_{s,t}\subset O_{s,t}$.

Since (S,V) and (T,U) are compact, the product space is also compact. On the other hand, we have $\underset{(s,t)\in S\times T}{⋃}V_{s,t}\times U_{s,t}=S\times T$, so ${\left\{V_{s,t}\times U_{s,t}\right\}}_{(s,t)\in S\times T}$ is an open cover of S×T. Therefore, there exist $s_{1},\ldots ,s_{n}\in S$ and $t_{1},\ldots ,t_{n}\in T$ such that $\overset{n}{\underset{i=1}{⋃}}V_{s_{i},t_{i}}\times U_{s_{i},t_{i}}=S\times T$.

Now, fix s∈S, and define $V_{s}=\underset{\begin{array}{c} 1\leq i\leq n,\\ s\in V_{s_{i},t_{i}} \end{array}}{⋂}V_{s_{i},t_{i}}$. Since $V_{s}$ is the intersection of finitely many open sets containing *s*, $V_{s}$ is an open neighborhood of *s* in (S,V). Let $s^{\prime }\in V_{s}$ and t∈T. Since $\overset{n}{\underset{i=1}{⋃}}V_{s_{i},t_{i}}\times U_{s_{i},t_{i}}=S\times T$, there exists 1≤i≤n such that $\left(s,t\right)\in V_{s_{i},t_{i}}\times U_{s_{i},t_{i}}\subset O_{s_{i},t_{i}}$. Since $s\in V_{s_{i},t_{i}}$, we have $V_{s}\subset V_{s_{i},t_{i}}$, and so, $s^{\prime }\in V_{s_{i},t_{i}}$. Therefore, $\left(s^{\prime },t\right)\in V_{s_{i},t_{i}}\times U_{s_{i},t_{i}}\subset O_{s_{i},t_{i}}$, hence:

$$|f\left(s^{\prime },t\right)-f\left(s,t\right)|\leq |f\left(s^{\prime },t\right)-f\left(s_{i},t_{i}\right)|+|f\left(s_{i},t_{i}\right)-f\left(s,t\right)|<\frac{\epsilon }{2}+\frac{\epsilon }{2}=\epsilon .$$

However, this is true for every t∈T. Therefore,

$$\underset{t\in T}{\sup}\left|f\left(s^{\prime },t\right)-f\left(s,t\right)\right|\leq \epsilon .$$

## Appendix B. Continuity of the Product of Measures

For every subset *A* of $M_{1}\times M_{2}$ and every $x_{1}\in M_{1}$, define $A_{2}^{x_{1}}=\left\{x_{2}\in M_{2}:\;\left(x_{1},x_{2}\right)\in A\right\}$. Similarly, for every $x_{2}\in M_{2}$, define $A_{1}^{x_{2}}=\left\{x_{1}\in M_{1}:\;\left(x_{1},x_{2}\right)\in A\right\}$. Let $P_{1},P_{1}^{\prime }\in P\left(M_{1},\Sigma_{1}\right)$ and $P_{2},P_{2}^{\prime }\in P\left(M_{2},\Sigma_{2}\right)$. We have:

$$\begin{array}{cc} ∥P_{1}\times P_{2}- & P_{1}^{\prime }\times P_{2}^{\prime }{∥}_{TV}=\underset{A\in \Sigma_{1}⊗\Sigma_{2}}{\sup}\left|\left(P_{1}\times P_{2}\right)\left(A\right)-\left(P_{1}^{\prime }\times P_{2}^{\prime }\right)\left(A\right)\right|\\ & \leq \underset{A\in \Sigma_{1}⊗\Sigma_{2}}{\sup}\left\{|\left(P_{1}\times P_{2}\right)\left(A\right)-\left(P_{1}^{\prime }\times P_{2}\right)\left(A\right)|+|\left(P_{1}^{\prime }\times P_{2}\right)\left(A\right)-\left(P_{1}^{\prime }\times P_{2}^{\prime }\right)\left(A\right)|\right\}\\ & =\underset{A\in \Sigma_{1}⊗\Sigma_{2}}{\sup}\{\left|\int_{M_{2}}P_{1}\left(A_{1}^{x_{2}}\right)\cdot dP_{2}\left(x_{2}\right)-\int_{M_{2}}P_{1}^{\prime }\left(A_{1}^{x_{2}}\right)\cdot dP_{2}\left(x_{2}\right)\right|\\ & \;\;\;\;\;\;\;\;\;\;\;\;\;\;\;\;\;\;\;\;\;\;\;\;\;\;+\left|\int_{M_{1}}P_{2}\left(A_{2}^{x_{1}}\right)\cdot dP_{1}^{\prime }\left(x_{1}\right)-\int_{M_{1}}P_{2}^{\prime }\left(A_{2}^{x_{1}}\right)\cdot dP_{1}^{\prime }\left(x_{1}\right)\right|\}\\ & \leq \underset{A\in \Sigma_{1}⊗\Sigma_{2}}{\sup}\left\{\int_{M_{2}}\left|P_{1}\left(A_{1}^{x_{2}}\right)-P_{1}^{\prime }\left(A_{1}^{x_{2}}\right)\right|\cdot dP_{2}\left(x_{2}\right)+\int_{M_{1}}\left|P_{2}\left(A_{2}^{x_{1}}\right)-P_{2}^{\prime }\left(A_{2}^{x_{1}}\right)\right|\cdot dP_{1}^{\prime }\left(x_{1}\right)\right\}\\ & \leq \int_{M_{2}}\left(\underset{A_{1}\in \Sigma_{1}}{\sup}\left|P_{1}\left(A_{1}\right)-P_{1}^{\prime }\left(A_{1}\right)\right|\right)dP_{2}+\int_{M_{1}}\left(\underset{A_{2}\in \Sigma_{2}}{\sup}\left|P_{2}\left(A_{2}\right)-P_{2}^{\prime }\left(A_{2}\right)\right|\right)dP_{1}^{\prime }\\ & =∥P_{1}-P_{1}^{\prime }{∥}_{TV}+{∥P_{2}-P_{2}^{\prime }∥}_{TV}. \end{array}$$

This shows that the product of measures is continuous under the total variation topology.

## Appendix C. Proof of Proposition 1

Define the mapping $G:M\to R^{+}\cup \left\{+\infty \right\}$ as follows:

$$G\left(x\right)=\int_{M^{\prime }}g\left(y\right)d\left(R\left(x\right)\right)\left(y\right).$$

For every n≥0, define the mapping $g_{n}:M^{\prime }\to R^{+}$ as follows:

$$g_{n}\left(y\right)=\frac{1}{2^{n}}\left\lfloor 2^{n}\times \min\left\{n,g\left(y\right)\right\}\right\rfloor .$$

Clearly, for every $y\in M^{\prime }$ we have:

- $g_{n}\left(y\right)\leq g\left(y\right)$ for all n≥0.
- $g_{n}\left(y\right)\leq g_{n+1}\left(y\right)$ for all n≥0.
- $\lim_{n\to \infty }g_{n}\left(y\right)=g\left(y\right)$.

Moreover, for every fixed n≥0, we have:

- $g_{n}$ is $\Sigma^{\prime }$-measurable.
- $g_{n}$ takes values in $\left\{\frac{i}{2^{n}}:\;0\leq i\leq n2^{n}\right\}$.

For every $0\leq i\leq n2^{n}$, let $B_{i,n}=\left\{y\in M^{\prime }:\;g_{n}\left(y\right)=\frac{i}{2^{n}}\right\}$. Since $g_{n}$ is $\Sigma^{\prime }$-measurable, we have $B_{i,n}\in \Sigma^{\prime }$ for every $0\leq i\leq n2^{n}$. Now, for every n≥0, define the mapping $G_{n}:M\to R\cup \left\{+\infty \right\}$ as follows:

$$\begin{array}{cc} G_{n}\left(x\right) & =\int_{M^{\prime }}g_{n}\left(y\right)d\left(R\left(x\right)\right)\left(y\right)=\int_{M^{\prime }}\left(\sum_{i=0}^{n2^{n}}\frac{i}{2^{n}}{𝟙}_{B_{i,n}}\left(y\right)\right)d\left(R\left(x\right)\right)\left(y\right)\\ & =\sum_{i=0}^{n2^{n}}\frac{i}{2^{n}}\left(R\left(x\right)\right)\left(B_{i,n}\right)=\sum_{i=0}^{n2^{n}}\frac{i}{2^{n}}R_{B_{i,n}}\left(x\right). \end{array}$$

Since the random mapping *R* is measurable and since $B_{i,n}\in \Sigma^{\prime }$, the mapping $R_{B_{i,n}}$ is Σ-measurable for every $0\leq i\leq n2^{n}$. Therefore, $G_{n}$ is Σ-measurable for every n≥0. Moreover, for every x∈Σ, we have:

$$\begin{array}{cc} \lim_{n\to \infty }G_{n}\left(x\right) & =\lim_{n\to \infty }\int_{M^{\prime }}g_{n}\left(y\right)d\left(R\left(x\right)\right)\left(y\right)\overset{\left(a\right)}{=}\int_{M^{\prime }}g\left(y\right)d\left(R\left(x\right)\right)\left(y\right)=G\left(x\right), \end{array}$$

where (a) follows from the monotone convergence theorem. We conclude that *G* is Σ-measurable because it is the point-wise limit of Σ-measurable functions. On the other hand, we have:

$$\begin{array}{cc} \int_{M^{\prime }}g_{n}\cdot d\left(R_{\#}P\right) & =\sum_{i=0}^{n2^{n}}\frac{i}{2^{n}}\left(R_{\#}P\right)\left(B_{i,n}\right)=\sum_{i=0}^{n2^{n}}\frac{i}{2^{n}}\int_{M}R_{B_{i,n}}\left(x\right)\cdot dP\left(x\right)\\ & =\sum_{i=0}^{n2^{n}}\frac{i}{2^{n}}\int_{M}\left(R\left(x\right)\right)\left(B_{i,n}\right)\cdot dP\left(x\right)=\sum_{i=0}^{n2^{n}}\frac{i}{2^{n}}\int_{M}\left(\int_{M^{\prime }}{𝟙}_{B_{i,n}}\left(y\right)\cdot d\left(R\left(x\right)\right)\left(y\right)\right)dP\left(x\right)\\ & =\int_{M}\left(\int_{M^{\prime }}\left(\sum_{i=0}^{n2^{n}}\frac{i}{2^{n}}{𝟙}_{B_{i,n}}\left(y\right)\right)d\left(R\left(x\right)\right)\left(y\right)\right)dP\left(x\right)\\ & =\int_{M}\left(\int_{M^{\prime }}g_{n}\left(y\right)d\left(R\left(x\right)\right)\left(y\right)\right)dP\left(x\right)=\int_{M}G_{n}\cdot dP. \end{array}$$

Therefore,

$$\int_{M^{\prime }}g\cdot d\left(R_{\#}P\right)\overset{\left(a\right)}{=}\lim_{n\to \infty }\int_{M^{\prime }}g_{n}\cdot d\left(R_{\#}P\right)=\lim_{n\to \infty }\int_{M}G_{n}\cdot dP\overset{\left(b\right)}{=}\int_{M}G\cdot dP,$$

where (a) and (b) follow from the monotone convergence theorem.

## Appendix D. Continuity of the Push-Forward by a Random Mapping

Let *R* be a measurable random mapping from (M,Σ) to $\left(M^{\prime },\Sigma^{\prime }\right)$. Let $P_{1},P_{2}\in P\left(M,\Sigma \right)$. Define the signed measure $\mu =P_{1}-P_{2}$, and let $\left\{\mu^{+},\mu^{-}\right\}$ be the Jordan measure decomposition of μ. It is easy to see that $∥P_{1}-P_{2}{∥}_{TV}=\mu^{+}\left(M\right)=\mu^{-}\left(M\right)$. For every $B\in \Sigma^{\prime }$, we have:

$$\begin{array}{cc} \left(R_{\#}\left(P_{1}\right)\right)\left(B\right)-\left(R_{\#}\left(P_{2}\right)\right)\left(B\right) & =\int_{M}R_{B}\cdot dP_{1}-\int_{M}R_{B}\cdot dP_{2}=\int_{M}R_{B}\cdot d\left(P_{1}-P_{2}\right)\\ & =\int_{M}R_{B}\cdot d\left(\mu^{+}-\mu^{-}\right)\leq \int_{M}R_{B}\cdot d\mu^{+}\leq {∥R_{B}∥}_{\infty }\cdot \mu^{+}\left(M\right)\\ & \overset{\left(a\right)}{\leq }\mu^{+}\left(M\right)={∥P_{1}-P_{2}∥}_{TV}, \end{array}$$

where (a) follows from the fact that $|R_{B}\left(x\right)|=|\left(R\left(x\right)\right)\left(B\right)|\leq 1$ for every x∈M. We can similarly show that:

$$\left(R_{\#}\left(P_{2}\right)\right)\left(B\right)-\left(R_{\#}\left(P_{1}\right)\right)\left(B\right)\leq ∥R_{B}{∥}_{\infty }\cdot \mu^{-}\left(M\right)\leq {∥P_{1}-P_{2}∥}_{TV}.$$

Therefore,

$$\begin{array}{c} ∥R_{\#}\left(P_{1}\right)-R_{\#}\left(P_{2}\right){∥}_{TV}=\underset{B\in \Sigma^{\prime }}{\sup}|\left(R_{\#}\left(P_{1}\right)\right)\left(B\right)-\left(R_{\#}\left(P_{2}\right)\right)\left(B\right)|\leq ∥P_{1}-P_{2}{∥}_{TV}. \end{array}$$

This shows that the push-forward mapping $R_{\#}$ from P(M,Σ) to $P(M^{\prime },\Sigma^{\prime })$ is continuous under the total variation topology. This concludes the proof of Lemma 3.

Now, assume that U is a Polish topology on *M* and $U^{\prime }$ is an arbitrary topology on $M^{\prime }$. Let *R* be measurable random mapping from (M,B(M)) to $\left(M^{\prime },B\left(M^{\prime }\right)\right)$. Moreover, assume that *R* is a continuous mapping from (M,U) to $P(M^{\prime },B\left(M^{\prime }\right))$ when the latter space is endowed with the weak-∗ topology. Let ${\left(P_{n}\right)}_{n\geq 0}$ be a sequence of probability measures in P(M,B(M)) that weakly-∗ converges to P∈P(M,B(M)).

Let $g:M^{\prime }\to R$ be a bounded and continuous mapping. Define the mapping G:M→R as follows:

$$G\left(x\right)=\int_{M^{\prime }}g\left(y\right)\cdot d\left(R\left(x\right)\right)\left(y\right).$$

For every sequence ${\left(x_{n}\right)}_{n\geq 0}$ converging to *x* in *M*, the sequence ${\left(R\left(x_{n}\right)\right)}_{n\geq 0}$ weakly-∗ converges to R(x) in $P(M^{\prime },B\left(M^{\prime }\right))$ because of the continuity of *R*. This implies that the sequence ${\left(G\left(x_{n}\right)\right)}_{n\geq 0}$ converges to G(x). Since U is a Polish topology (hence, metrizable and sequential [20]), this shows that *G* is a bounded and continuous mapping from (M,U) to R. Therefore, we have:

$$\begin{array}{c} \lim_{n\to \infty }\int_{M^{\prime }}g\cdot d\left(R_{\#}P_{n}\right)\overset{\left(a\right)}{=}\lim_{n\to \infty }\int_{M}G\cdot dP_{n}\overset{\left(b\right)}{=}\int_{M}G\cdot dP\overset{\left(c\right)}{=}\int_{M^{\prime }}g\cdot d\left(R_{\#}P\right), \end{array}$$

where (a) and (c) follow from Corollary 2, and (b) follows from the fact that ${\left(P_{n}\right)}_{n\geq 0}$ weakly-∗ converges to *P*. This shows that ${\left(R_{\#}P_{n}\right)}_{n\geq 0}$ weakly-∗ converges to $R_{\#}P$. Now, since U is Polish, the weak-∗ topology on P(M,B(M)) is metrizable [21]; hence, it is sequential [20]. This shows that the push-forward mapping $R_{\#}$ from P(M,B(M)) to $P(M^{\prime },B\left(M^{\prime }\right))$ is continuous under the weak-∗ topology.

## Appendix E. Proof of Lemma 5

For every s∈S, define the mapping $f_{s}:\Delta_{X}\to R$ as $f_{s}\left(p\right)=f\left(s,p\right)$. Clearly $f_{s}$ is continuous for every s∈S. Therefore, the mapping $F_{s}:\mathrm{MP}\left(X\right)\to R$ defined as:

$$F_{s}\left(\mathrm{MP}\right)=\int_{\Delta_{X}}f_{s}\cdot d\mathrm{MP}$$

is continuous in the weak-∗ topology of MP(X).

Fix ϵ>0, and let (s,MP)∈S×MP(X). Since $F_{s}$ is continuous, there exists a weakly-∗ open neighborhood $U_{s,\mathrm{MP}}$ of MP such that $|F_{s}\left({\mathrm{MP}}^{\prime }\right)-F_{s}\left(\mathrm{MP}\right)|<\frac{\epsilon }{2}$ for every ${\mathrm{MP}}^{\prime }\in U_{s,\mathrm{MP}}$. On the other hand, Lemma 1 implies the existence of an open neighborhood $V_{s}$ of *s* in (S,V) such that for every $s^{\prime }\in V_{s}$, we have:

$$\underset{p\in \Delta_{X}}{\sup}\left|f\left(s^{\prime },p\right)-f\left(s,p\right)\right|\leq \frac{\epsilon }{2}.$$

Clearly $V_{s}\times U_{s,\mathrm{MP}}$ is an open neighborhood of (s,MP) in S×MP(X). For every $\left(s^{\prime },{\mathrm{MP}}^{\prime }\right)\in V_{s}\times U_{s,\mathrm{MP}}$, we have:

$$\begin{array}{cc} |F\left(s^{\prime },{\mathrm{MP}}^{\prime }\right)-F\left(s,\mathrm{MP}\right)| & \leq |F\left(s^{\prime },{\mathrm{MP}}^{\prime }\right)-F\left(s,{\mathrm{MP}}^{\prime }\right)|+|F\left(s,{\mathrm{MP}}^{\prime }\right)-F\left(s,\mathrm{MP}\right)|\\ & =\left|\int_{\Delta_{X}}\left(f\left(s^{\prime },p\right)-f\left(s,p\right)\right)\cdot d{\mathrm{MP}}^{\prime }\left(p\right)\right|+\left|F_{s}\left({\mathrm{MP}}^{\prime }\right)-F_{s}\left(\mathrm{MP}\right)\right|\\ & <\left(\int_{\Delta_{X}}\left|f\left(s^{\prime },p\right)-f\left(s,p\right)\right|\cdot d{\mathrm{MP}}^{\prime }\left(p\right)\right)+\frac{\epsilon }{2}\overset{\left(a\right)}{\leq }\frac{\epsilon }{2}+\frac{\epsilon }{2}=\epsilon , \end{array}$$

where (a) follows from the fact that ${\mathrm{MP}}^{\prime }$ is a meta-probability measure and $|f\left(s^{\prime },p\right)-f\left(s^{\prime },p\right)|\leq \frac{\epsilon }{2}$ for every $p\in \Delta_{X}$. We conclude that *F* is continuous.

## Appendix F. Weak-∗ Continuity of the Product of Meta-Probability Measures

Let ${\left({\mathrm{MP}}_{1,n}\right)}_{n\geq 0}$ and ${\left({\mathrm{MP}}_{2,n}\right)}_{n\geq 0}$ be two sequences that weakly-∗ converge to ${\mathrm{MP}}_{1}$ and ${\mathrm{MP}}_{2}$ in $\mathrm{MP}(X_{1})$ and $\mathrm{MP}(X_{2})$, respectively. Let $f:\Delta_{X_{1}}\times \Delta_{X_{2}}\to R$ be a continuous and bounded mapping. Define the mapping $F:\Delta_{X_{1}}\times \mathrm{MP}\left(X_{2}\right)$ as follows:

$$F\left(p_{1},{\mathrm{MP}}_{2}^{\prime }\right)=\int_{\Delta_{X_{2}}}f\left(p_{1},p_{2}\right)d{\mathrm{MP}}_{2}^{\prime }\left(p_{2}\right).$$

Fix ϵ>0. Since $f(p_{1},p_{2})$ is continuous, Lemma 5 implies that *F* is continuous. Therefore, the mapping $p_{1}\to F\left(p_{1},{\mathrm{MP}}_{2}\right)$ is continuous on $\Delta_{X_{1}}$, which implies that it is also bounded because $\Delta_{X_{1}}$ is compact. Therefore,

$$\lim_{n\to \infty }\int_{\Delta_{X_{1}}}F\left(p_{1},{\mathrm{MP}}_{2}\right)d{\mathrm{MP}}_{1,n}\left(p_{1}\right)=\int_{\Delta_{X_{1}}}F\left(p_{1},{\mathrm{MP}}_{2}\right)d{\mathrm{MP}}_{1}\left(p_{1}\right)$$

because ${\left({\mathrm{MP}}_{1,n}\right)}_{n\geq 0}$ weakly-∗ converges to ${\mathrm{MP}}_{1}$. This means that there exists $n_{1}\geq 0$ such that for every $n\geq n_{1}$, we have:

$$\left|\int_{\Delta_{X_{1}}}F\left(p_{1},{\mathrm{MP}}_{2}\right)d{\mathrm{MP}}_{1,n}\left(p_{1}\right)-\int_{\Delta_{X_{1}}}F\left(p_{1},{\mathrm{MP}}_{2}\right)d{\mathrm{MP}}_{1}\left(p_{1}\right)\right|<\frac{\epsilon }{2}.$$

On the other hand, since *F* is continuous and since $\mathrm{MP}(X_{2})$ is compact under the weak-∗ topology [21], Lemma 1 implies the existence of a weakly-∗ open neighborhood $U_{{\mathrm{MP}}_{2}}$ of ${\mathrm{MP}}_{2}$ such that $|F\left(p_{1},{\mathrm{MP}}_{2}^{\prime }\right)-F\left(p_{1},{\mathrm{MP}}_{2}\right)|\leq \frac{\epsilon }{2}$ for every ${\mathrm{MP}}_{2}^{\prime }\in U_{{\mathrm{MP}}_{2}}$ and every $p_{1}\in \Delta_{X_{1}}$. Moreover, since ${\mathrm{MP}}_{2,n}$ weakly-∗ converges to ${\mathrm{MP}}_{2}$, there exists $n_{2}\geq 0$ such that ${\mathrm{MP}}_{2,n}\in U_{{\mathrm{MP}}_{2}}$ for every $n\geq n_{2}$.

Therefore, for every $n\geq \max\{n_{1},n_{2}\}$, we have:

$$\begin{array}{c} \left|\int_{\Delta_{X_{1}}}\left(\int_{\Delta_{X_{2}}}f\left(p_{1},p_{2}\right)d{\mathrm{MP}}_{2,n}\left(p_{2}\right)\right)d{\mathrm{MP}}_{1,n}\left(p_{1}\right)-\int_{\Delta_{X_{1}}}\left(\int_{\Delta_{X_{2}}}f\left(p_{1},p_{2}\right)d{\mathrm{MP}}_{2}\left(p_{2}\right)\right)d{\mathrm{MP}}_{1}\left(p_{1}\right)\right|\\ \leq \left|\int_{\Delta_{X_{1}}}\left(\int_{\Delta_{X_{2}}}f\left(p_{1},p_{2}\right)d{\mathrm{MP}}_{2,n}\left(p_{2}\right)\right)d{\mathrm{MP}}_{1,n}\left(p_{1}\right)-\int_{\Delta_{X_{1}}}\left(\int_{\Delta_{X_{2}}}f\left(p_{1},p_{2}\right)d{\mathrm{MP}}_{2}\left(p_{2}\right)\right)d{\mathrm{MP}}_{1,n}\left(p_{1}\right)\right|\\ \;\;+\left|\int_{\Delta_{X_{1}}}\left(\int_{\Delta_{X_{2}}}f\left(p_{1},p_{2}\right)d{\mathrm{MP}}_{2}\left(p_{2}\right)\right)d{\mathrm{MP}}_{1,n}\left(p_{1}\right)-\int_{\Delta_{X_{1}}}\left(\int_{\Delta_{X_{2}}}f\left(p_{1},p_{2}\right)d{\mathrm{MP}}_{2}\left(p_{2}\right)\right)d{\mathrm{MP}}_{1}\left(p_{1}\right)\right|\\ =\left|\int_{\Delta_{X_{1}}}\left(F\left(p_{1},{\mathrm{MP}}_{2,n}\right)-F\left(p_{1},{\mathrm{MP}}_{2}\right)\right)d{\mathrm{MP}}_{1,n}\left(p_{1}\right)\right|\\ \;\;\;\;\;\;\;\;\;\;\;\;\;\;\;\;\;\;\;\;\;\;\;\;\;\;\;\;\;\;\;\;\;\;\;\;\;\;\;\;\;\;\;\;\;\;\;\;\;\;\;\;\;\;\;\;+\left|\int_{\Delta_{X_{1}}}F\left(p_{1},{\mathrm{MP}}_{2}\right)d{\mathrm{MP}}_{1,n}\left(p_{1}\right)-\int_{\Delta_{X_{1}}}F\left(p_{1},{\mathrm{MP}}_{2}\right)d{\mathrm{MP}}_{1}\left(p_{1}\right)\right|\\ <\int_{\Delta_{X_{1}}}\left|F\left(p_{1},{\mathrm{MP}}_{2,n}\right)-F\left(p_{1},{\mathrm{MP}}_{2}\right)\right|d{\mathrm{MP}}_{1,n}\left(p_{1}\right)+\frac{\epsilon }{2}\overset{\left(a\right)}{\leq }\int_{\Delta_{X_{1}}}\frac{\epsilon }{2}\cdot d{\mathrm{MP}}_{1,n}\left(p_{1}\right)+\frac{\epsilon }{2}=\epsilon , \end{array}$$

where (a) follows from the fact ${\mathrm{MP}}_{2,n}\in U_{{\mathrm{MP}}_{2}}$ for every $n\geq n_{2}$. Therefore,

$$\begin{array}{cc} \lim_{n\to \infty }\int_{\Delta_{X_{1}}\times \Delta_{X_{2}}}f\cdot d\left({\mathrm{MP}}_{1,n}\times {\mathrm{MP}}_{2,n}\right) & \overset{\left(a\right)}{=}\lim_{n\to \infty }\int_{\Delta_{X_{1}}}\left(\int_{\Delta_{X_{2}}}f\left(p_{1},p_{2}\right)d{\mathrm{MP}}_{2,n}\left(p_{2}\right)\right)d{\mathrm{MP}}_{1,n}\left(p_{1}\right)\\ & =\int_{\Delta_{X_{1}}}\left(\int_{\Delta_{X_{2}}}f\left(p_{1},p_{2}\right)d{\mathrm{MP}}_{2}\left(p_{2}\right)\right)d{\mathrm{MP}}_{1}\left(p_{1}\right)\\ & \overset{\left(b\right)}{=}\int_{\Delta_{X_{1}}\times \Delta_{X_{2}}}f\cdot d\left({\mathrm{MP}}_{1}\times {\mathrm{MP}}_{2}\right), \end{array}$$

where (a) and (b) follow from Fubini’s theorem. We conclude that ${\left({\mathrm{MP}}_{1,n}\times {\mathrm{MP}}_{2,n}\right)}_{n\geq 0}$ weakly-∗ converges to ${\left({\mathrm{MP}}_{1}\times {\mathrm{MP}}_{2}\right)}_{n\geq 0}$. Therefore, the product of meta-probability measures is weakly-∗ continuous.

## Appendix G. Continuity of the Capacity

Since the mapping *I* is continuous and since the space $\Delta_{X}\times {\mathrm{DMC}}_{X,Y}$ is compact, the mapping *I* is uniformly continuous, i.e., for every ϵ>0, there exists δ(ϵ)>0 such that for every $\left(p_{1},W_{1}\right),\left(p_{2},W_{2}\right)\in \Delta_{X}\times {\mathrm{DMC}}_{X,Y}$, if $∥p_{1}-p_{2}{∥}_{1}:=\sum_{x\in X}\left|p_{1}\left(x\right)-p_{2}\left(x\right)\right|<\delta \left(\epsilon \right)$ and $d_{X,Y}\left(W_{1},W_{2}\right)<\delta \left(\epsilon \right)$, then

$$|I\left(p_{1},W_{1}\right)-I\left(p_{2},W_{2}\right)|<\epsilon .$$

Let $W_{1},W_{2}\in {\mathrm{DMC}}_{X,Y}$ be such that $d_{X,Y}\left(W_{1},W_{2}\right)<\delta \left(\epsilon \right)$. For every $p\in \Delta_{X}$, we have ${∥p-p∥}_{1}=0<\delta \left(\epsilon \right)$, so we must have $|I\left(p,W_{1}\right)-I\left(p,W_{2}\right)|<\epsilon$. Therefore,

$$\begin{array}{c} I\left(p,W_{1}\right)<I\left(p,W_{2}\right)+\epsilon \leq \underset{p^{\prime }\in \Delta_{X}}{\sup}I\left(p^{\prime },W_{2}\right)+\epsilon =C\left(W_{2}\right)+\epsilon . \end{array}$$

Therefore,

$$\begin{array}{c} C\left(W_{1}\right)=\underset{p\in \Delta_{X}}{\sup}I\left(p,W_{1}\right)\leq C\left(W_{2}\right)+\epsilon . \end{array}$$

Similarly, we can show that $C\left(W_{2}\right)\leq C\left(W_{1}\right)+\epsilon$. This implies that $|C\left(W_{1}\right)-C\left(W_{2}\right)|\leq \epsilon$; hence, *C* is continuous.

## Appendix H. Measurability and Continuity of *C*^+,∗^

Let us first show that the random mapping $C^{+,*}$ is measurable. We need to show that the mapping $C_{B}^{+,*}:\Delta_{X}\times \Delta_{X}\to R$ is measurable for every $B\in B(\Delta_{X})$, where:

$$C_{B}^{+,*}\left(p_{1},p_{2}\right)=\left(C^{+,*}\left(p_{1},p_{2}\right)\right)\left(B\right),\;\;\forall p_{1},p_{2}\in \Delta_{X}.$$

For every $u_{1}\in X$, define the set:

$$A_{u_{1}}=\left\{\left(p_{1},p_{2}\right)\in \Delta_{X}\times \Delta_{X}:\;\left(C^{-,*}\left(p_{1},p_{2}\right)\right)\left(u_{1}\right)>0\right\}.$$

Clearly, $A_{u_{1}}$ is open in $\Delta_{X}\times \Delta_{X}$ (and so it is measurable). The mapping $C^{+,u_{1},*}$ is defined on $A_{u_{1}}$, and it is clearly continuous. Therefore, for every $B\in B(\Delta_{X})$, ${\left(C^{+,u_{1},*}\right)}^{-1}\left(B\right)$ is measurable. We have:

$$\begin{array}{cc} C_{B}^{+,*}\left(p_{1},p_{2}\right) & =\left(C^{+,*}\left(p_{1},p_{2}\right)\right)\left(B\right)=\sum_{\begin{array}{c} u_{1}\in \mathrm{supp}\left(C^{-,*}\left(p_{1},p_{2}\right)\right),\\ C^{+,u_{1},*}\left(p_{1},p_{2}\right)\in B \end{array}}\left(C^{-,*}\left(p_{1},p_{2}\right)\right)\left(u_{1}\right)\\ & =\sum_{\begin{array}{c} u_{1}\in X,\\ \left(p_{1},p_{2}\right)\in A_{u_{1}},\\ C^{+,u_{1},*}\left(p_{1},p_{2}\right)\in B \end{array}}\left(C^{-,*}\left(p_{1},p_{2}\right)\right)\left(u_{1}\right)\overset{\left(a\right)}{=}\sum_{u_{1}\in X}\left(C^{-,*}\left(p_{1},p_{2}\right)\right)\left(u_{1}\right)\cdot {𝟙}_{{\left(C^{+,u_{1},*}\right)}^{-1}\left(B\right)}\left(p_{1},p_{2}\right), \end{array}$$

where (a) follows from the fact that $\left(p_{1},p_{2}\right)\in {\left(C^{+,u_{1},*}\right)}^{-1}\left(B\right)$ if and only if $\left(p_{1},p_{2}\right)\in A_{u_{1}}$ and $C^{+,u_{1},*}\left(p_{1},p_{2}\right)\in B$. This shows that $C_{B}^{+,*}$ is measurable for every $B\in B(\Delta_{X})$. Therefore, $C^{+,*}$ is a measurable random mapping.

Let ${\left(p_{1,n},p_{2,n}\right)}_{n\geq 0}$ be a converging sequence to $\left(p_{1},p_{2}\right)$ in $\Delta_{X}\times \Delta_{X}$. Since $C^{-,*}$ is continuous, we have $\lim_{n\to \infty }\left(C^{-,*}\left(p_{1,n},p_{2,n}\right)\right)\left(u_{1}\right)=\left(C^{-,*}\left(p_{1},p_{2}\right)\right)\left(u_{1}\right)$ for every $u_{1}\in X$. Therefore, for every $u_{1}\in \mathrm{supp}\left(C^{-,*}\left(p_{1},p_{2}\right)\right)$, there exists $n_{u_{1}}\geq 0$ such that for every $n\geq n_{u_{1}}$, we have $C^{-,*}\left(p_{1,n},p_{2,n}\right)>0$. Let $n_{0}=\max\left\{n_{u_{1}}:\;u_{1}\in \mathrm{supp}\left(C^{-,*}\left(p_{1},p_{2}\right)\right)\right\}$. For every $n\geq n_{0}$, we have $\mathrm{supp}\left(C^{-,*}\left(p_{1},p_{2}\right)\right)\subset \mathrm{supp}\left(C^{-,*}\left(p_{1,n},p_{2,n}\right)\right)$. Therefore, for every continuous and bounded mapping $g:\Delta_{X}\to R$, we have:

$$\begin{array}{cc} \lim_{n\to \infty }\int_{\Delta_{X}}g\cdot d\left(C^{+,*}\left(p_{1,n},p_{2,n}\right)\right) & =\lim_{n\to \infty }\sum_{u_{1}\in \mathrm{supp}\left(C^{-,*}\left(p_{1,n},p_{2,n}\right)\right)}g\left(C^{+,u_{1},*}\left(p_{1,n},p_{2,n}\right)\right)\cdot \left(C^{-,*}\left(p_{1,n},p_{2,n}\right)\right)\left(u_{1}\right)\\ & \overset{\left(a\right)}{=}\lim_{n\to \infty }\sum_{u_{1}\in \mathrm{supp}\left(C^{-,*}\left(p_{1},p_{2}\right)\right)}g\left(C^{+,u_{1},*}\left(p_{1,n},p_{2,n}\right)\right)\cdot \left(C^{-,*}\left(p_{1,n},p_{2,n}\right)\right)\left(u_{1}\right)\\ & \overset{\left(b\right)}{=}\sum_{u_{1}\in \mathrm{supp}\left(C^{-,*}\left(p_{1},p_{2}\right)\right)}g\left(C^{+,u_{1},*}\left(p_{1},p_{2}\right)\right)\cdot \left(C^{-,*}\left(p_{1},p_{2}\right)\right)\left(u_{1}\right)\\ & =\int_{\Delta_{X}}g\cdot d\left(C^{+,*}\left(p_{1},p_{2}\right)\right), \end{array}$$

where (b) follows from the continuity of *g* and $C^{-,*}$ and the continuity of $C^{+,u_{1},*}$ on $A_{u_{1}}$ for every $u_{1}\in X$. (a) follows from the fact that:

$$\begin{array}{cc} \lim_{n\to \infty }\sum_{\begin{array}{c} u_{1}\in \mathrm{supp}\left(C^{-,*}\left(p_{1,n},p_{2,n}\right)\right),\\ u_{1}\notin \mathrm{supp}\left(C^{-,*}\left(p_{1},p_{2}\right)\right) \end{array}} & \left|g\left(C^{+,u_{1},*}\left(p_{1,n},p_{2,n}\right)\right)\cdot \left(C^{-,*}\left(p_{1,n},p_{2,n}\right)\right)\left(u_{1}\right)\right|\\ & \leq {∥g∥}_{\infty }\lim_{n\to \infty }\sum_{\begin{array}{c} u_{1}\in \mathrm{supp}\left(C^{-,*}\left(p_{1,n},p_{2,n}\right)\right),\\ u_{1}\notin \mathrm{supp}\left(C^{-,*}\left(p_{1},p_{2}\right)\right) \end{array}}\left(C^{-,*}\left(p_{1,n},p_{2,n}\right)\right)\left(u_{1}\right)\\ & ={∥g∥}_{\infty }\lim_{n\to \infty }\left(1-\sum_{u_{1}\in \mathrm{supp}\left(C^{-,*}\left(p_{1},p_{2}\right)\right)}\left(C^{-,*}\left(p_{1,n},p_{2,n}\right)\right)\left(u_{1}\right)\right)\\ & ={∥g∥}_{\infty }\left(1-\sum_{u_{1}\in \mathrm{supp}\left(C^{-,*}\left(p_{1},p_{2}\right)\right)}\left(C^{-,*}\left(p_{1},p_{2}\right)\right)\left(u_{1}\right)\right)=0. \end{array}$$

We conclude that the mapping $C^{+,*}$ is a continuous mapping from $\Delta_{X}\times \Delta_{X}$ to MP(X) when the latter space is endowed with the weak-∗ topology.

## Appendix I. Proof of Proposition 10

Let ${\hat{W}}_{1}\in {\mathrm{DMC}}_{X_{1},*}^{\left(o\right)}$ and ${\bar{W}}_{2}\in {\mathrm{DMC}}_{X_{2},*}^{\left(o\right)}$. Fix $W_{1}\in {\hat{W}}_{1}$ and $W_{2}\in {\bar{W}}_{2}$, and let $Y_{1}$ and $Y_{2}$ be the output alphabets of $W_{1}$ and $W_{2}$, respectively. We may assume without loss of generality that Im(W1)=Y1 and Im(W2)=Y2.

Let $y\in Y_{1}$. We have:

$$\begin{array}{cc} P_{W_{1}⊕W_{2}}^{o}\left(y\right) & =\frac{1}{|X_{1}∐X_{2}|}\sum_{x\in X_{1}∐X_{2}}\left(W_{1}⊕W_{2}\right)\left(y|x\right)\\ & =\frac{1}{|X_{1}\left|+\right|X_{2}|}\sum_{x\in X_{1}}W_{1}\left(y|x\right)=\frac{|X_{1}|}{|X_{1}\left|+\right|X_{2}|}P_{W_{1}}^{o}\left(y\right)>0. \end{array}$$

For every $x\in X_{1}$, we have:

$${\left(W_{1}⊕W_{2}\right)}_{y}^{-1}\left(x\right)=\frac{\left(W_{1}⊕W_{2}\right)\left(y|x\right)}{\left(\right|X_{1}\left|+\right|X_{2}\left|\right)P_{W_{1}}^{o}\left(y\right)}=\frac{W_{1}\left(y|x\right)}{|X_{1}|P_{W_{1}}^{o}\left(y\right)}={\left(W_{1}\right)}_{y}^{-1}\left(x\right).$$

On the other hand, for every $x\in X_{2}$, we have:

$${\left(W_{1}⊕W_{2}\right)}_{y}^{-1}\left(x\right)=\frac{\left(W_{1}⊕W_{2}\right)\left(y|x\right)}{\left(\right|X_{1}\left|+\right|X_{2}\left|\right)P_{W_{1}}^{o}\left(y\right)}=0.$$

Therefore, ${\left(W_{1}⊕W_{2}\right)}_{y}^{-1}=\phi_{1\#}{\left(W_{1}\right)}_{y}^{-1}$, where $\phi_{1}$ is the canonical injection from $X_{1}$ to $X_{1}∐X_{2}$.

Similarly, for every $y\in Y_{2}$, we have $P_{W_{1}⊕W_{2}}^{o}\left(y\right)=\frac{|X_{2}|}{|X_{1}\left|+\right|X_{2}|}P_{W_{1}}^{o}\left(y\right)>0$ and ${\left(W_{1}⊕W_{2}\right)}_{y}^{-1}=\phi_{2\#}{\left(W_{2}\right)}_{y}^{-1}$, where $\phi_{2}$ is the canonical injection from $X_{2}$ to $X_{1}∐X_{2}$. For every $B\in B(\Delta_{X_{1}∐X_{2}})$, we have:

$$\begin{array}{cc} {\mathrm{MP}}_{W_{1}⊕W_{2}}\left(B\right) & =\sum_{\begin{array}{c} y\in Y_{1}∐Y_{2},\\ {\left(W_{1}⊕W_{2}\right)}_{y}^{-1}\in B \end{array}}P_{W_{1}⊕W_{2}}^{o}\left(y\right)\\ & =\left(\sum_{\begin{array}{c} y\in Y_{1},\\ \phi_{1\#}{\left(W_{1}\right)}_{y}^{-1}\in B \end{array}}\frac{|X_{1}|}{|X_{1}\left|+\right|X_{2}|}P_{W_{1}}^{o}\left(y\right)\right)+\left(\sum_{\begin{array}{c} y\in Y_{2},\\ \phi_{2\#}{\left(W_{2}\right)}_{y}^{-1}\in B \end{array}}\frac{|X_{2}|}{|X_{1}\left|+\right|X_{2}|}P_{W_{2}}^{o}\left(y\right)\right)\\ & =\frac{|X_{1}|}{|X_{1}\left|+\right|X_{2}|}{\mathrm{MP}}_{W_{1}}\left({\left(\phi_{1\#}\right)}^{-1}\left(B\right)\right)+\frac{|X_{2}|}{|X_{1}\left|+\right|X_{2}|}{\mathrm{MP}}_{W_{2}}\left({\left(\phi_{2\#}\right)}^{-1}\left(B\right)\right)\\ & =\frac{|X_{1}|}{|X_{1}\left|+\right|X_{2}|}\left(\phi_{1\#\#}{\mathrm{MP}}_{W_{1}}\right)\left(B\right)+\frac{|X_{2}|}{|X_{1}\left|+\right|X_{2}|}\left(\phi_{2\#\#}{\mathrm{MP}}_{W_{2}}\right)\left(B\right). \end{array}$$

Therefore,

$${\mathrm{MP}}_{{\hat{W}}_{1}⊕{\bar{W}}_{2}}=\frac{|X_{1}|}{|X_{1}\left|+\right|X_{2}|}\phi_{1\#\#}{\mathrm{MP}}_{{\hat{W}}_{1}}+\frac{|X_{2}|}{|X_{1}\left|+\right|X_{2}|}\phi_{2\#\#}{\mathrm{MP}}_{{\bar{W}}_{2}}.$$

This shows the first formula of Proposition 10.

For every $y=\left(y_{1},y_{2}\right)\in Y_{1}\times Y_{2}$, we have:

$$\begin{array}{cc} P_{W_{1}⊗W_{2}}^{o}\left(y\right) & =\sum_{\left(x_{1},x_{2}\right)\in X_{1}\times X_{2}}\frac{1}{|X_{1}\times X_{2}|}\left(W_{1}⊗W_{2}\right)\left(y_{1},y_{2}|x_{1},x_{2}\right)\\ & =\sum_{\begin{array}{c} x_{1}\in X_{2},\\ x_{2}\in X_{2} \end{array}}\frac{W_{1}\left(y_{1}|x_{1}\right)}{|X_{1}|}\cdot \frac{W_{2}\left(y_{2}|x_{2}\right)}{|X_{2}|}=P_{W_{1}}^{o}\left(y_{1}\right)P_{W_{2}}^{o}\left(y_{2}\right)>0. \end{array}$$

For every $x=\left(x_{1},x_{2}\right)\in X_{1}\times X_{2}$, we have:

$$\begin{array}{cc} {\left(W_{1}⊗W_{2}\right)}_{y}^{-1}\left(x\right) & =\frac{\left(W_{1}⊗W_{2}\right)\left(y|x\right)}{|X_{1}\times X_{2}|P_{W_{1}⊗W_{2}}^{o}\left(y\right)}=\frac{W_{1}\left(y_{1}|x_{1}\right)}{|X_{1}|P_{W_{1}}^{o}\left(y_{1}\right)}\cdot \frac{W_{2}\left(y_{2}|x_{2}\right)}{|X_{2}|P_{W_{2}}^{o}\left(y_{2}\right)}\\ & ={\left(W_{1}\right)}_{y_{1}}^{-1}\left(x_{1}\right)\cdot {\left(W_{2}\right)}_{y_{2}}^{-1}\left(x_{2}\right)=\left({\left(W_{1}\right)}_{y_{1}}^{-1}\times {\left(W_{2}\right)}_{y_{2}}^{-1}\right)\left(x\right). \end{array}$$

For every $B\in B(\Delta_{X_{1}\times X_{2}})$, we have:

$$\begin{array}{cc} {\mathrm{MP}}_{W_{1}⊗W_{2}}\left(B\right) & =\sum_{\begin{array}{c} y\in Y_{1}\times Y_{2},\\ {\left(W_{1}⊗W_{2}\right)}_{y}^{-1}\in B \end{array}}P_{W_{1}⊗W_{2}}^{o}\left(y\right)=\sum_{\begin{array}{c} y\in Y_{1}\times Y_{2},\\ {\left(W_{1}\right)}_{y_{1}}^{-1}\times {\left(W_{2}\right)}_{y_{2}}^{-1}\in B \end{array}}P_{W_{1}}^{o}\left(y_{1}\right)P_{W_{2}}^{o}\left(y_{2}\right)\\ & =\sum_{\begin{array}{c} y\in Y_{1}\times Y_{2},\\ \mathrm{Mul}\left({\left(W_{1}\right)}_{y_{1}}^{-1},{\left(W_{2}\right)}_{y_{2}}^{-1}\right)\in B \end{array}}P_{W_{1}}^{o}\left(y_{1}\right)P_{W_{2}}^{o}\left(y_{2}\right)=\left({\mathrm{MP}}_{W_{1}}\times {\mathrm{MP}}_{W_{2}}\right)\left({\mathrm{Mul}}^{-1}\left(B\right)\right)\\ & =\left({\mathrm{Mul}}_{\#}\left({\mathrm{MP}}_{W_{1}}\times {\mathrm{MP}}_{W_{2}}\right)\right)\left(B\right)=\left({\mathrm{MP}}_{W_{1}}⊗{\mathrm{MP}}_{W_{2}}\right)\left(B\right). \end{array}$$

Therefore,

$${\mathrm{MP}}_{{\hat{W}}_{1}⊗{\bar{W}}_{2}}={\mathrm{MP}}_{{\hat{W}}_{1}}⊗{\mathrm{MP}}_{{\bar{W}}_{2}}.$$

This shows the second formula of Proposition 10.

Now, let α∈[0,1] and ${\hat{W}}_{1},{\hat{W}}_{2}\in {\mathrm{DMC}}_{X,*}^{\left(o\right)}$. Fix $W_{1}\in {\hat{W}}_{1}$ and $W_{2}\in {\hat{W}}_{2}$, and let $Y_{1}$ and $Y_{2}$ be the output alphabets of $W_{1}$ and $W_{2}$, respectively. We may assume without loss of generality that Im(W1)=Y1 and Im(W2)=Y2. Let $W=[\alpha W_{1},\left(1-\alpha \right)W_{2}]$. If α=0, then *W* is equivalent to $W_{2}$ and ${\mathrm{MP}}_{W}={\mathrm{MP}}_{W_{2}}=\alpha {\mathrm{MP}}_{W_{1}}+\left(1-\alpha \right){\mathrm{MP}}_{W_{2}}$. If α=1, then *W* is equivalent to $W_{1}$ and ${\mathrm{MP}}_{W}={\mathrm{MP}}_{W_{1}}=\alpha {\mathrm{MP}}_{W_{1}}+\left(1-\alpha \right){\mathrm{MP}}_{W_{2}}$.

Assume now that 0<α<1. For every $y\in Y_{1}$, we have:

$$\begin{array}{c} P_{W}^{o}\left(y\right)=\frac{1}{\left|X\right|}\sum_{x\in X}W\left(y|x\right)=\frac{1}{\left|X\right|}\sum_{x\in X}\alpha \cdot W_{1}\left(y|x\right)=\alpha P_{W_{1}}^{o}\left(y\right)>0. \end{array}$$

For every x∈X, we have:

$$\begin{array}{c} W_{y}^{-1}\left(x\right)=\frac{W(y|x)}{\left|X\right|P_{W}^{o}\left(y\right)}=\frac{\alpha W_{1}\left(y|x\right)}{|X|\alpha P_{W_{1}}^{o}\left(y\right)}={\left(W_{1}\right)}_{y}^{-1}\left(x\right). \end{array}$$

Similarly, for every $y\in Y_{2}$, we have $P_{W}^{o}\left(y\right)=\left(1-\alpha \right)P_{W_{2}}^{o}\left(y\right)>0$ and $W_{y}^{-1}={\left(W_{2}\right)}_{y}^{-1}$. Therefore,

$$\begin{array}{cc} {\mathrm{MP}}_{W} & =\sum_{y\in Y_{1}∐Y_{2}}P_{W}^{o}\left(y\right)\cdot \delta_{W_{y}^{-1}}=\left(\sum_{y\in Y_{1}}\alpha P_{W_{1}}^{o}\left(y\right)\cdot \delta_{{\left(W_{1}\right)}_{y}^{-1}}\right)+\left(\sum_{y\in Y_{2}}\left(1-\alpha \right)P_{W_{2}}^{o}\left(y\right)\cdot \delta_{{\left(W_{2}\right)}_{y}^{-1}}\right)\\ & =\alpha {\mathrm{MP}}_{W_{1}}+\left(1-\alpha \right){\mathrm{MP}}_{W_{2}}. \end{array}$$

Therefore,

$${\mathrm{MP}}_{\left[\alpha {\hat{W}}_{1},\left(1-\alpha \right){\hat{W}}_{2}\right]}=\alpha {\mathrm{MP}}_{{\hat{W}}_{1}}+\left(1-\alpha \right){\mathrm{MP}}_{{\hat{W}}_{2}}.$$

This shows the third formula of Proposition 10.

Now, let $\hat{W}\in {\mathrm{DMC}}_{X,*}^{\left(o\right)}$, and let ∗ be a uniformity preserving binary operation on X. Fix $W\in \hat{W}$, and let Y be the output alphabet of *W*. We may assume without loss of generality that Im(W)=Y.

Let $U_{1},U_{2}$ be two independent random variables uniformly distributed in X. Let $X_{1}=U_{1}*U_{2}$ and $X_{2}=U_{2}$. Send $X_{1}$ and $X_{2}$ through two independent copies of *W*, and let $Y_{1}$ and $Y_{2}$ be the output, respectively.

For every $\left(y_{1},y_{2}\right)\in Y^{2}$, we have:

$$P_{W^{-}}^{o}\left(y_{1},y_{2}\right)=P_{Y_{1},Y_{2}}\left(y_{1},y_{2}\right)=P_{Y_{1}}\left(y_{1}\right)P_{Y_{2}}\left(y_{2}\right)=P_{W}^{o}\left(y_{1}\right)P_{W}^{o}\left(y_{2}\right)>0.$$

For every $u_{1}\in X$, we have:

$$\begin{array}{cc} {\left(W^{-}\right)}_{y_{1},y_{2}}^{-1}\left(u_{1}\right) & =P_{U_{1}|Y_{1},Y_{2}}\left(u_{1}|y_{1},y_{2}\right)=\sum_{u_{2}\in X_{2}}P_{U_{1},U_{2}|Y_{1},Y_{2}}\left(u_{1},u_{2}|y_{1},y_{2}\right)\\ & =\sum_{u_{2}\in X_{2}}P_{X_{1},X_{2}|Y_{1},Y_{2}}\left(u_{1}*u_{2},u_{2}|y_{1},y_{2}\right)=\sum_{u_{2}\in X_{2}}P_{X_{1}|Y_{1}}\left(u_{1}*u_{2}|y_{1}\right)P_{X_{2}|Y_{2}}\left(u_{2}|y_{2}\right)\\ & =\sum_{u_{2}\in X_{2}}W_{y_{1}}^{-1}\left(u_{1}*u_{2}\right)W_{y_{2}}^{-1}\left(u_{2}\right)=\left(C^{-,*}\left(W_{y_{1}}^{-1},W_{y_{2}}^{-1}\right)\right)\left(u_{1}\right). \end{array}$$

For every $B\in B(\Delta_{X})$, we have:

$$\begin{array}{cc} {\mathrm{MP}}_{W^{-}}\left(B\right) & =\sum_{\begin{array}{c} y\in Y^{2},\\ {\left(W^{-}\right)}_{y}^{-1}\in B \end{array}}P_{W^{-}}^{o}\left(y\right)=\sum_{\begin{array}{c} \left(y_{1},y_{2}\right)\in Y^{2},\\ C^{-,*}\left(W_{y_{1}}^{-1},W_{y_{2}}^{-1}\right)\in B \end{array}}P_{W_{1}}^{o}\left(y_{1}\right)P_{W_{2}}^{o}\left(y_{2}\right)\\ & =\left({\mathrm{MP}}_{W}\times {\mathrm{MP}}_{W}\right)\left({\left(C^{-,*}\right)}^{-1}\left(B\right)\right)=\left(C_{\#}^{-,*}\left({\mathrm{MP}}_{W}\times {\mathrm{MP}}_{W}\right)\right)\left(B\right)={\left({\mathrm{MP}}_{W},{\mathrm{MP}}_{W}\right)}^{-,*}\left(B\right). \end{array}$$

Therefore,

$${\mathrm{MP}}_{{\hat{W}}^{-}}={\left({\mathrm{MP}}_{\hat{W}},{\mathrm{MP}}_{\hat{W}}\right)}^{-,*}.$$

This shows the forth formula of Proposition 10.

For every $\left(y_{1},y_{2},u_{1}\right)\in Y^{2}\times X$, we have:

$$\begin{array}{cc} P_{W^{+}}^{o}\left(y_{1},y_{2},u_{1}\right) & =P_{Y_{1},Y_{2},U_{1}}\left(y_{1},y_{2},y_{1}\right)=P_{Y_{1},Y_{2}}\left(y_{1},y_{2}\right)P_{U_{1}|Y_{1},Y_{2}}\left(u_{1}|y_{1},y_{2}\right)\\ & =P_{W}^{o}\left(y_{1}\right)P_{W}^{o}\left(y_{2}\right)\cdot \left(C^{-,*}\left(W_{y_{1}}^{-1},W_{y_{2}}^{-1}\right)\right)\left(u_{1}\right). \end{array}$$

Therefore,

Im(W+)=⋃(y1,y2)∈Y2{(y1,y2)}×supp(C−,∗(Wy1−1,Wy2−1)).

For every (y1,y2,u1)∈Im(W+), we have:

$$\begin{array}{cc} {\left(W^{+}\right)}_{y_{1},y_{2},u_{1}}^{-1}\left(u_{2}\right) & =P_{U_{2}|Y_{1},Y_{2},U_{1}}\left(u_{2}|y_{1},y_{2},u_{1}\right)=\frac{P_{U_{1},U_{2}|Y_{1},Y_{2}}\left(u_{1},u_{2}|y_{1},y_{2}\right)}{P_{U_{1}|Y_{1},Y_{2}}\left(u_{1}|y_{1},y_{2}\right)}\\ & =\frac{P_{X_{1}|Y_{1}}\left(u_{1}*u_{2}|y_{1}\right)P_{X_{2}|Y_{2}}\left(u_{2}|y_{2}\right)}{\left(C^{-,*}\left(W_{y_{1}}^{-1},W_{y_{2}}^{-1}\right)\right)\left(u_{1}\right)}=\frac{W_{y_{1}}^{-1}\left(u_{1}*u_{2}\right)W_{y_{2}}^{-1}\left(u_{2}\right)}{\left(C^{-,*}\left(W_{y_{1}}^{-1},W_{y_{2}}^{-1}\right)\right)\left(u_{1}\right)}\\ & =\left(C^{+,u_{1},*}\left(W_{y_{1}}^{-1},W_{y_{2}}^{-1}\right)\right)\left(u_{2}\right). \end{array}$$

For every $B\in B(\Delta_{X})$, we have:

$$\begin{array}{cc} {\mathrm{MP}}_{W^{+}}\left(B\right) & =\sum_{\left(y_{1},y_{2}\right)\in Y^{2}}\sum_{\begin{array}{c} u_{1}\in \mathrm{supp}(C^{-,*}\left(W_{y_{1}}^{-1},W_{y_{2}}^{-1}\right),\\ C^{+,u_{1},*}\left(W_{y_{1}}^{-1},W_{y_{2}}^{-1}\right)\in B \end{array}}P_{W}^{o}\left(y_{1}\right)P_{W}^{o}\left(y_{2}\right)\cdot \left(C^{-,*}\left(W_{y_{1}}^{-1},W_{y_{2}}^{-1}\right)\right)\left(u_{1}\right)\\ & =\sum_{\left(y_{1},y_{2}\right)\in Y^{2}}P_{W}^{o}\left(y_{1}\right)P_{W}^{o}\left(y_{2}\right)\sum_{\begin{array}{c} u_{1}\in \mathrm{supp}(C^{-,*}\left(W_{y_{1}}^{-1},W_{y_{2}}^{-1}\right),\\ C^{+,u_{1},*}\left(W_{y_{1}}^{-1},W_{y_{2}}^{-1}\right)\in B \end{array}}\left(C^{-,*}\left(W_{y_{1}}^{-1},W_{y_{2}}^{-1}\right)\right)\left(u_{1}\right)\\ & =\sum_{\left(y_{1},y_{2}\right)\in Y^{2}}P_{W}^{o}\left(y_{1}\right)P_{W}^{o}\left(y_{2}\right)\left(C^{+,*}\left(W_{y_{1}}^{-1},W_{y_{2}}^{-1}\right)\right)\left(B\right)\\ & =\sum_{\left(y_{1},y_{2}\right)\in Y^{2}}P_{W}^{o}\left(y_{1}\right)P_{W}^{o}\left(y_{2}\right)(C_{B}^{+,*}\left(W_{y_{1}}^{-1},W_{y_{2}}^{-1}\right)\\ & =\int_{\Delta_{X}\times \Delta_{X}}C_{B}^{+,*}\left(p_{1},p_{2}\right)\cdot d\left({\mathrm{MP}}_{W}\times {\mathrm{MP}}_{W}\right)\left(p_{1},p_{2}\right)\\ & =\left(C_{\#}^{+,*}\left({\mathrm{MP}}_{W}\times {\mathrm{MP}}_{W}\right)\right)\left(B\right)={\left({\mathrm{MP}}_{W},{\mathrm{MP}}_{W}\right)}^{+,*}\left(B\right). \end{array}$$

Therefore,

$${\mathrm{MP}}_{{\hat{W}}^{+}}={\left({\mathrm{MP}}_{\hat{W}},{\mathrm{MP}}_{\hat{W}}\right)}^{+,*}.$$

This shows the fifth and last formula of Proposition 10.

## References

[1] Nasser R. Continuity of Channel Parameters and Operations under Various DMC Topologies. Proceedings of the IEEE International Symposium on Information Theory (ISIT).

[2] Polyanskiy Y., Poor H.V., Verdu S. (2010). Channel Coding Rate in the Finite Blocklength Regime. IEEE Trans. Inf. Theory.

[3] Polyanskiy Y. (2013). Saddle Point in the Minimax Converse for Channel Coding. IEEE Trans. Inf. Theory.

[4] Schwarte H. (1996). On weak convergence of probability measures, channel capacity and code error probabilities. IEEE Trans. Inf. Theory.

[5] Richardson T., Urbanke R. (2008). Modern Coding Theory.

[6] Nasser R. (2017). Topological Structures on DMC spaces. arXiv.

[7] Engelking R. (1977). General Topology.

[8] Schieler C., Cuff P. (2016). The Henchman Problem: Measuring Secrecy by the Minimum Distortion in a List. IEEE Trans. Inf. Theory.

[9] Torgersen E. (1991). Comparison of Statistical Experiments.

[10] Şaşoğlu E., Telatar E., Arıkan E. Polarization for Arbitrary Discrete Memoryless Channels. Proceedings of the IEEE Information Theory Workshop.

[11] Nasser R. (2017). An Ergodic Theory of Binary Operations, Part II: Applications to Polarization. IEEE Trans. Inf. Theory.

[12] Cover T., Thomas J. (2006). Elements of Information Theory.

[13] Shannon C. (1956). The zero error capacity of a noisy channel. IRE Trans. Inf. Theory.

[14] Mondelli M., Hassani S.H., Urbanke R.L. (2014). From Polar to Reed-Muller Codes: A Technique to Improve the Finite-Length Performance. IEEE Trans. Inf. Theory.

[15] Arıkan E. (2009). Channel Polarization: A Method for Constructing Capacity-Achieving Codes for Symmetric Binary-Input Memoryless Channels. IEEE Trans. Inf. Theory.

[16] Shannon C. (1958). A Note on a Partial Ordering for Communication Channels. Inform. Contr..

[17] Steenrod N.E. (1967). A convenient category of topological spaces. Michigan Math. J..

[18] Nasser R. (2016). An Ergodic Theory of Binary Operations, Part I: Key Properties. IEEE Trans. Inf. Theory.

[19] Raginsky M. Channel Polarization and Blackwell Measures. Proceedings of the IEEE International Symposium on Information Theory (ISIT).

[20] Franklin S. (1965). Spaces in which sequences suffice. Fundam. Math..

[21] Villani C. (2003). Topics in Optimal Transportation.

